# No evidence that selection is resource-demanding in conflict and bilingual language production tasks: Implications for theories of adaptive control and language-control associations

**DOI:** 10.3758/s13423-025-02672-y

**Published:** 2025-03-07

**Authors:** Giacomo Spinelli, Simone Sulpizio

**Affiliations:** Department of Psychology, University of Milano-Bicocca, Piazza Dell’Ateneo Nuovo 1, 20126 Milan, Italy

**Keywords:** Adaptive control, Cognitive control, Bilingualism, Bilingual advantage

## Abstract

Theories of adaptive (and cognitive) control assume that selecting target information in the context of highly salient distractors depends on limited-capacity resources. Building on this assumption, theories of language-control associations propose that the opportunities afforded by bilingualism to engage such effortful selection, such as when speaking in a nondominant language, might improve domain-general adaptive control. The assumption that domain-general or language-specific selection is resource-demanding, however, has surprisingly little empirical support. Here, we tested that assumption by having unbalanced Italian-English bilinguals perform both an L1 Stroop task and an L2 picture-naming task simultaneously with an *n*-back task. Both tasks showed costs due to the load produced by the *n*-back task and distractor interference, with slower responses to incongruent (the word GREEN in the color red) *and* congruent stimuli (RED in red) than neutral ones (XXX in red) in the L1 Stroop task and to noncognate than cognate pictures (pictures with different/similar L1 and L2 names) in the L2 picture-naming task. However, neither task showed larger distractor interference with greater load, with Bayesian analyses favoring the absence of such interactions. These results suggest that domain-general and language-specific selection may occur reactively, i.e., only when the need arises, with no strong reliance on limited-capacity resources. Further, they invite a rethinking of both adaptive-control theories assuming resource-demanding selection and theories of language-control associations assuming that regularly engaging such selection would be conducive to domain-general benefits.

**Supplementary Information:**

The online version contains supplementary material available at 10.3758/s13423-025-02672-y.

## Introduction

It is not unusual for research programs, including some so popular as to inspire other research programs, to turn out to rest on little or weak empirical support. The present research highlighted one such example concerning adaptive control. Adaptive control refers to the ability to modulate certain aspects of control (e.g., selection – the ability to respond to a color ignoring a word in a conflict task such as the Stroop (1935) task) in order to accommodate the current context (as created, e.g., by a manipulation of conflict frequency; Braem et al., 2019). According to domain-general theories of adaptive control such as the dual mechanisms of control framework (Braver, 2012), adaptive control would manifest itself, among other situations, when selection is tightened in contexts that favor a *proactive* control mode in which conflict from distractors is anticipated (an efficient mode, but demanding in terms of limited-capacity attentional resources) versus relaxed in contexts that favor a *reactive* mode in which such conflict is only addressed when it occurs (a less efficient but also less resource-demanding mode).

Consistent with this and similar accounts (e.g., Botvinick et al., 2001; Kane & Engle, 2003), the Stroop effect (i.e., the latency difference between incongruent stimuli, e.g., RED in blue, and congruent stimuli, e.g., RED in red) is typically smaller in a mostly incongruent list (a list assumed to induce proactive control) than in a mostly congruent list (a list assumed to induce reactive control; Logan & Zbrodoff, 1979; for a demonstration of the robustness of the effect, see Spinelli & Lupker, 2023a). Surprisingly, however, the empirical record is not as strong for manipulations that should be, on paper, particularly effective as they test a foundational assumption of adaptive-control theories – that selection is resource-demanding under normal circumstances, i.e., when proactive control can be engaged. In these manipulations, a Stroop(-like) task is performed concurrently with another task, typically a working-memory task of varying difficulty creating a load on attentional resources (e.g., de Fockert et al., 2001). The rationale is that proactive control is possible when the secondary task produces a low load but not when it produces a high load consuming most of the available resources. With reactive control being enforced instead, not only should an overall processing cost emerge but so should a larger Stroop effect reflecting the more relaxed state of selection associated with reactive control.

While some researchers reported this interaction (de Fockert et al., 2001; Lavie et al., 2004), others failed to do so, at least when focusing on the basic incongruent-congruent contrast (Soutschek et al., 2013; Spinelli et al., 2020; Suh & Bugg, 2021). On the other hand, Kalanthroff et al. (2015) reported an experiment with the potential to solve these apparent inconsistencies. Reasoning that congruent and incongruent stimuli, while conveying non-conflicting versus conflicting *information*, both evoke conflicting *tasks* (i.e., the reading task in addition to the color-naming task), Kalanthroff et al. added a neutral unreadable condition which did not involve that task conflict. With that addition, they found the typical Stroop pattern in a low-load list – a large interference effect (incongruent – neutral) and a small, but positive, facilitation effect (neutral – congruent). Notably, however, in a high-load list, a reverse facilitation effect emerged, as congruent stimuli were *slower* than neutral ones. This pattern would seem to suggest that the main impact of load manipulations on selection might be an increased cost to task conflict. Such an increase would have little impact on the incongruent-congruent contrast but, in the neutral-congruent contrast, would be capable of eliminating and even reversing the facilitation effect congruent stimuli normally produce compared to neutral stimuli.

However, this conclusion might be premature because of a number of methodological issues in Kalanthroff et al.’s experiment. We present those issues in the *Method* section, along with a Stroop experiment conducted in participants’ dominant language (i.e., their L1) which fixes them and should, therefore, provide a clearer picture of whether resource availability does play a role in selection as adaptive-control theories assume. The point, for now, is that there is little empirical support for such a foundational assumption.

A similar case can be made for a class of theories which have been inspired by adaptive-control theories – theories of language-control associations, such as the bilingual-advantage hypothesis, which propose that regular experience managing two languages would grant bilinguals an advantage (or a change) in cognitive control relative to monolinguals (Bialystok et al., 2004). Recently, those theories have elected adaptive (or attentional) control as the core driver of language-control associations (Bialystok, 2017; Green & Abutalebi, 2013; see also Bialystok, 2024). However, most of the empirical effort concerning those theories has been directed to testing their predictions (i.e., the existence of bilingual-monolingual differences; Paap, 2022) rather than their assumptions (i.e., that language control does involve adaptive control; Antoniou, 2019; Blanco-Elorrieta & Caramazza, 2021).

A potential exception is represented by studies which have examined the overlap between language-specific and domain-general control, typically using language-switching paradigms for the former and task-switching paradigms for the latter (e.g., Branzi et al., 2016; Calabria et al., 2012; Declerck et al., 2017, 2021; Weissberger et al., 2015). While the two paradigms produced some noteworthy differences (e.g., Calabria et al., 2012), their patterns largely overlapped. This is hardly surprising given that language switching intrinsically involves some form of domain-general control (Festman & Schwieter, 2015), especially when cued, as in most of those studies (Blanco-Elorrieta & Pylkkänen, 2018). Further, the notion of adaptive control is typically studied using not switching but *conflict* paradigms such as the Stroop task (Braem et al., 2019). Therefore, a test of the assumption that language control involves adaptive control would seem to require a contrast between that type of paradigm and a bilingual analog of it, with domain-general control being involved in that analog not because it is “built in” the paradigm but because of how the relevant processes are envisioned.

An important motivation for hypothesizing language-control associations has been the idea that when producing the name of a concept in a language – the target language – conflict will arise from the corresponding name in the other language – the non-target language – which, albeit irrelevant, will be active at the same time (e.g., Green, 1998). This conflict would be especially relevant when the non-target language is the bilingual’s L1 (with the target language being their L2; e.g., Hermans et al., 1998) and the two translation equivalents have very different pronunciations (i.e., they are noncognates; e.g., an Italian-English bilingual saying “horse” while ignoring “cavallo,” its Italian equivalent). In contrast, conflict will be reduced (and facilitation will arise) when the translation equivalents have similar pronunciations (i.e., they are cognates, e.g., “elephant” and “elefante”). The finding that pictures with cognate names are named faster than pictures with noncognate names, especially in the L2, is consistent with this idea (Costa et al., 2000). Since conflict is a basic premise of the theories of language-control associations we aim to test (Bialystok, 2017; Green & Abutalebi, 2013), it would seem appropriate to conduct that test using the cognate effect as a bilingual analog of the Stroop effect (see also Spinelli & Sulpizio, 2024). Both effects would reflect the relative inability of selecting a target (L2 name/color) when a more salient distractor (L1 name/word) is inevitably processed (because of automatic L1 activation/word reading), either helping (cognate/congruent) or hindering selection (noncognate/incongruent).

Under these premises, just like the size of the Stroop effect is an index of domain-general selection, the size of the cognate effect would be an index of bilingual selection. Further, under the assumption that the latter, just like the former, is under adaptive control, it follows from the logic discussed above for domain-general adaptive control that bilingual selection should suffer when proactive control cannot be engaged. Specifically, applying a load manipulation should have a parallel impact on the cognate effect as it should on Stroop effects: Not only should an overall processing cost emerge with high load but the cognate effect should increase because, with reactive control being enforced, selection should be less efficient. Such an interaction would support the assumption of theories of language-control associations that adaptive control is involved in bilingualism; an additive pattern, on the contrary, would challenge it. We tested this idea in an L2 picture-naming experiment modelled after the L1 Stroop experiment based on Kalanthroff et al. (2015) discussed above.

## Method

### Participants

The determination of the sample size was based on a power analysis conducted with G*Power 3.1 (Faul et al., 2009) using the effect size of the interaction between congruency and load in the Stroop task reported by Kalanthroff et al. (2015), ${\upeta }_{\text{p}}^{2}$ = 0.292. Although the minimum sample size suggested by the analysis for a 0.80 power to detect that effect size in Kalanthroff et al.’s design was 15, we aimed to reach a sample size comparable to that used in Spinelli and Sulpizio’s (2024) study, i.e., 48 participants. Participants were recruited from the same pool (i.e., the University of Milano-Bicocca community) and in the same fashion described by Spinelli and Sulpizio (2024; participants who already participated in the studies therein described were prevented from participating in the present study). Participants received course credits for their participation. To participate, volunteers were required to consider Italian to be their native (or one of their native) language(s), to have normal or corrected-to-normal vision and hearing, to be between 18 and 45 years old, and to pass an English pre-screening test (see below). 119 participants completed the pre-screening test. Of these, 67 passed it, 56 came to the lab to complete the study, and 48 remained after exclusions (see below). Of the final sample, 32 identified themselves as female and 16 male; they were 22.77 years old on average (*SD* = 3.90, range = 19–39 years); 40 reported knowing a third language beside Italian and English and 18 a fourth language, although their proficiency, immersion, and dominance in those languages (as calculated using the Language History Questionnaire (LHQ3); Li et al., 2019) were lower than those reported for Italian or English on average; all were born in Italy except three who came to live in Italy during childhood; and all resided in Italy. We report additional information on Italian (L1) and English (L2), the two languages involved in the study, in Table 1.

**Table 1:** Characteristics of Italian (L1) and English (L2) for our participants

	Italian (L1)			English (L2)		
Characteristic	Mean	SD	Range	Mean	SD	Range
Proficiency	0.97	0.06	0.71–1	0.81	0.09	0.57–1
Immersion	0.92	0.04	0.73–0.96	0.73	0.06	0.49–0.90
Dominance	0.61	0.06	0.48–0.75	0.46	0.07	0.33–0.65
Pre-screening score				21.46	2.06	18–25
Lexical fluency				61.85	11.29	39–85

Proficiency, immersion, and dominance are aggregated scores ranging from 0 to 1 calculated using the formulas in the LHQ3 (applying the corrections explained in Spinelli & Sulpizio, 2024; note that because of those corrections, dominance for the final sample could not be calculated for Italian in ten cases and for English in two cases; note further that proficiency could not be calculated for Italian for two participants who did not report Italian in response to the relevant questionnaire item). The pre-screening score is the sum of correct responses to the 25 questions included in Cambridge’s online test for adult learners of English, and lexical fluency is the number of correct L1-to-L2 translations provided for 90 words (see *Materials and procedur*e section).

### Materials and procedure

#### Pre-screening session

As in Spinelli and Sulpizio (2024), participants were pre-screened using Cambridge’s online test for adult learners of English (https://www.cambridgeenglish.org/test-your-english/general-english/). The test provides an English proficiency estimate within the Common European Framework of Reference for Languages (CEFRL). To pass the test, participants were required to perform at an estimated B2 CEFRL level. To participate in the pre-screening test and the subsequent lab session, participants expressed their informed consent. The study was evaluated by the local commission for minimal-risk studies of the Psychology Department at the University of Milano-Bicocca (protocol RM-2021–445).

#### Lab session

Participants who passed the pre-screening test were invited to participate in the lab session, which comprised a language background questionnaire, followed by an L1 Stroop experiment and an L2 picture-naming task presented in counterbalanced order across participants, followed by an L1-to-L2 translation task. The reason the order of L2 picture naming and L1 Stroop was counterbalanced was to control for potential practice effects for the load manipulation, which was common to both tasks. All instructions were given in Italian. The experimenter was in the room with the participant during all instructions and practice sessions and ensured that the participant understood each task providing additional explanations when necessary. The whole session took about 2.5 h to complete.

##### Language background questionnaire

To assess participants’ language background, we used the Language History Questionnaire 3.0 (LHQ3; Li et al., 2019), a validated tool to measure, by self-report, several aspects of the bilingual experience such as age of acquisition (AoA), proficiency, and patterns of language use. We used the same questionnaire as in Spinelli and Sulpizio (2024), an Italian translation of the English version presented using the Jotform (https://www.jotform.com/) survey services.

##### L1 Stroop experiment 

We used a combination of a Stroop and an *n*-back task similar to that of Kalanthroff et al. (2015) with the following exceptions. First, the experiment was conducted in Italian (our participants’ L1) rather than Hebrew (the L1 of Kalanthroff et al.’s participants). Second, we included slightly more trials in each load condition (192 vs. 180 in Kalanthroff et al.). Third, instead of requiring manual responses to colors as Kalanthroff et al. did, we required vocal responses, the gold standard in Stroop research (MacLeod, 1992). Indeed, because manual responses are typically arbitrary for colors, a manual color-word Stroop task cannot be considered a proper Stroop task but rather a Stroop-*like* task – a task type involving overlapping representations between targets (e.g., colors) and distractors (e.g., color names) but not between those stimulus components and responses (e.g., keypresses). In contrast, a vocal color-word Stroop task *can* be considered a proper Stroop task – a task type involving overlapping representations between targets, distractors, and responses (Kornblum, 1992; see also Viviani et al., 2024). Consistent with these ideas, different patterns of results have been reported for manual versus vocal Stroop tasks (e.g., Augustinova et al., 2019; Sharma & McKenna, 1998). Since the purpose of the Stroop task in the present research was to examine a domain-general phenomenon (i.e., adaptive control), it was important that the Stroop task be conducted in its standard configuration, i.e., with vocal responses. For the same reason, we conducted the task in participants’ L1, the language typically used in Stroop tasks, rather than their L2, which is known to produce a different pattern of results (e.g., Altarriba & Mathis, 1997; Tzelgov et al., 1990). Of course, it is possible that using either a manual L1 Stroop task or a vocal L2 Stroop task (or both options at the same time, i.e., a manual L2 Stroop task) might have produced a different result from that produced by our vocal L1 Stroop task. However, that result would have been attributable to either of those deviations from the standard Stroop configuration rather than reflecting a domain-general phenomenon. Future research should, however, explore those options.

The fourth and potentially most relevant difference with respect to Kalanthroff et al.’s experiment concerned the written stimuli we used in the Stroop task and the way in which they were combined with the colors. Similar to Kalanthroff et al., we used four colors (red, green, blue, and yellow) and the corresponding Italian names (ROSSO, VERDE, BLU, and GIALLO) to create the congruent and incongruent stimuli. To create the neutral stimuli, however, we used two letter strings, XXXXX and KKKKK, instead of one as in Kalanthroff et al. Further, whereas all of the written stimuli used in Kalanthroff et al.’s experiment were combined with all of the colors used, we split the written stimuli into two non-overlapping subsets: the words ROSSO, VERDE, and the letter string XXXXX, which were only presented in the colors red and green; and the words BLU, GIALLO, and the letter string KKKKK, which were only presented in the colors blue and yellow. The frequency of each color-word combination in each load condition in our experiment is presented in Table 2. Note that in both Kalanthroff et al. and the present experiment, each congruency condition involved 33% of the trials in each load condition.

**Table 2:** Frequency of color-word combinations in each of the load conditions in the L1 Stroop experiment

	Words					
Colors	ROSSO	VERDE	BLU	GIALLO	XXXXX	KKKKK
Red	16	16			16	
Green	16	16			16	
Blue			16	16		16
Yellow			16	16		16

The reason for these changes was to avoid a contingency-learning confound involved in Kalanthroff et al.’s (2015) design. Contingency learning refers to the finding that responding to a stimulus is easier if the response required is the typical one for the particular distractor involved in the stimulus (i.e., the high-contingency condition) and harder if the response is an atypical one for that distractor (i.e., the low-contingency condition) compared to when the stimulus involves a distractor with no typical/atypical response (i.e., the no-contingency condition; Lin & MacLeod, 2018; Schmidt et al., 2007). Crucially, Kalanthroff et al.’s (2015) design was such that *color name* distractors (i.e., the Hebrew translation equivalents of RED, GREEN, BLUE, and YELLOW) involved a typical response (i.e., the congruent response, such as the response “red” for the word RED, which was required on 50% of the trials in which RED was presented) and atypical responses (i.e., incongruent responses, such as the response “green” for the word RED, which was required on 16.67% of the trials in which RED was presented). In contrast, the *neutral letter string* distractor did not have a typical/atypical response as it appeared in each of the four colors on 25% of the trials in which it was presented. In other words, congruent stimuli were high-contingency stimuli, incongruent stimuli were low-contingency stimuli, and neutral stimuli were no-contingency stimuli. (In addition, whereas each of the color name distractors appeared on 30 trials in a block, the neutral letter string distractor appeared on 60 trials in a block, i.e., twice as frequently.)

The contingency-learning confound in Kalanthroff et al.’s design, albeit not atypical for Stroop experiments (Algom et al., 2022), is important for present purposes for two reasons (Sulpizio et al., 2024). First, it might have affected the pattern of the congruency effect at baseline, i.e., in the low-load condition. Specifically, the high-contingency nature of the congruent stimuli (vs. the no-contingency nature of the neutral stimuli) might have increased the facilitation effect (neutral – congruent). Similarly, the low-contingency nature of the incongruent stimuli might have increased the interference effect (incongruent – congruent). As a result of both of these changes, the Stroop effect itself (incongruent – congruent) would have also been increased. Note that it is not inevitable for such patterns, particularly the facilitation pattern, to emerge in designs involving no contingency-learning confound (Spinelli & Lupker, 2023b).

Second and most importantly, contingency learning is known to interact with load manipulations, with the difference between high- and low-contingency conditions becoming smaller under high load (Schmidt et al., 2010; Spinelli et al., 2020). Because congruency and contingency learning were confounded in Kalanthroff et al.’s (2015) design, the implication is that at least part of the congruency by load interaction effect they reported might have actually been driven by a *contingency* by load interaction. Specifically, whereas in the low-load condition, as explained, contingency learning would have increased the interference effect and, crucially, the facilitation effect, such might not have been possible in the high-load condition, a condition which might prevent contingencies from being learned and/or used (Schmidt et al., 2010). As a result, in the latter condition, there would have been little or no bias towards facilitation in the neutral-congruent contrast (or towards interference in the incongruent-neutral contrast). In sum, the contingency-learning confound might have favored the emergence of a congruency by load interaction in this type of design.

In contrast, there was no contingency-learning confound in our design (see Table 2). The reason is that, because each distractor (including both the color names and the letter strings) was presented equally frequently in two colors, there was no typical/atypical response for either congruent, incongruent, or neutral stimuli (i.e., all of the stimuli were no-contingency stimuli). As a result, our design did not intrinsically bias the emergence of a congruency by load interaction, even though our expectation, as noted, was still for such an interaction to emerge. (Note also that our design did not involve the distractor frequency confound mentioned for Kalanthroff et al.’s experiment either, as each color name distractor appeared on 32 trials in a block just as each letter string distractor did.)

Apart from these changes, the experiment was as close as possible to that of Kalanthroff et al. (2015). Specifically, in addition to the colored stimuli described (the stimuli relevant to the Stroop task), the experiment involved the letters B, D, G, P, and T (the stimuli relevant to the *n*-back task used for the load manipulation), each of which was presented an equivalent number of times across the practice and experimental trials within each load condition. In both load conditions, the trial sequence involved a fixation symbol ( +) presented for 500 ms, the letter stimulus presented for 1,150 ms (regardless of the participant’s response), another fixation symbol ( +) presented for 850 ms, the colored stimulus presented for 2,500 ms or until response, and a blank screen presented for 1,200 ms. All stimuli were presented in Courier New 14 point in the center of the screen against a medium-grey background. The order of presentation of the letter and colored stimuli within a block was randomized with the constraint that the two types of stimuli be presented in an alternated fashion, as described.

The two load conditions were administered in separate blocks. That is, the load manipulation was blocked, as in Kalanthroff et al. (2015) and, to the best of our knowledge, in any study using the *n*-back task. Note that a blocked load manipulation could help participants to develop strategies to deal with the particular load condition presented in the block, particularly with the high-load condition, thus reducing potential differences between that condition and the low-load one. Nonetheless, such differences emerge regularly in blocked load manipulations (e.g., Jonides et al., 1997), suggesting that whatever strategies the blocked presentation might enable are not completely effective at eliminating the differences between load levels. However, future research might consider using alternative tasks (e.g., simple short-term memory tasks: de Fockert et al., 2001; Lavie et al., 2004) which allow a mixed load manipulation.

The order of the low- and high-load block was counterbalanced across participants. This order, however, was always consistent with the order used for the L2 picture-naming experiment (e.g., participants who were presented with the low-load list first in that experiment were presented with the low-load list first in this experiment as well). In the instructions, participants were explained how to respond to the colored stimuli first and how to respond to the letter stimuli next. For the colored stimuli, in both load conditions, participants were instructed to name the color in Italian, their L1, as quickly and as accurately as possible. For the letter stimuli, in the low-load condition, one of the five letters used was chosen randomly for each participant and the participant was instructed to press “0” on the numpad with any finger of their right hand any time that target letter appeared (i.e., a 0-back task). In contrast, in the high-load condition, participants were instructed to respond to a letter (potentially, any letter) by pressing “0” only when that letter was identical to the letter presented two trials earlier (i.e., a 2-back task). Following Kalanthroff et al. (2015), we emphasized accuracy over speed for the *n*-back task, however, we also informed participants that they had slightly more than 1 s to make their response, should a response be required.

There was no feedback for either the Stroop task or the *n*-back task in the experiment proper. However, each load condition was preceded by a practice session which did involve feedback for misses and false alarms in the *n*-back task. The feedback message was an explanation of why participants should or should not have responded, depending on the situation, and remained on the screen until participants pressed “enter” on the numpad. For example, in the low-load condition for a participant assigned the letter T as the target letter, the feedback message was “You should not have pressed the button, because this letter was not a T” for a false alarm and “In this case, you should have pressed the button, because this letter was a T” for a miss (feedback messages were presented in Italian). In addition, the feedback message in the high-load condition reminded the participant what the last two presented letters were and invited participants to memorize those letters before resuming the practice session. The low-load condition included eight practice trials. The high-load condition also involved 8 practice trials for participants who performed the L2 picture-naming experiment first (and who, therefore, had had some experience with the 2-back task in the context of that experiment). Reasoning that participants who did not have any prior experience with the 2-back task would require more practice, the high-load condition for participants who performed the L1 Stroop experiment first involved 23 practice trials. DMDX (Forster & Forster, 2003) was used to program the experiment.

##### L2 picture-naming experiment 

We used the same picture stimuli used by Spinelli and Sulpizio (2024). They were 192 colored drawings, 96 with cognate and 96 with noncognate English and Italian names, selected from the MultiPic dataset (Duñabeitia et al., 2018) based on the results of a pilot study described in full, along with the selection process, in Spinelli and Sulpizio (2024). The most relevant characteristics are presented in Table 3. Note that cognate and noncognate stimuli differed widely on phonological similarity with Italian while being matched on picture visual complexity and word frequency. It was impossible to match cognate and noncognate words on length in syllables as well, because English words in an English-Italian cognate pair (typically words of Latin origin, e.g., “elephant” from Latin “elephantus”) tend to be longer than English words in an English-Italian noncognate pair (typically words of Germanic origin, e.g., “goat” from Old English “gat”; see, e.g., Bar-Ilan & Berman, 2007). However, the only impact such a mismatch could have is to reduce the cognate effect (for a detailed discussion, see Spinelli & Sulpizio, 2024). Because the aim of the present study is not to produce a completely confound-free cognate effect but to examine whether this effect would interact with the load manipulation, this particular mismatch does not pose a problem.

**Table 3:** Characteristics of the cognate and noncognate stimuli used in the L2 picture-naming experiment

	Cognate			Noncognate			*t*-test	
Characteristic	Mean	SD	Range	Mean	SD	Range	*t*	*p*
Visual complexity (picture)	2.46	0.46	1.19–3.45	2.36	0.32	1.42–3.39	1.53	0.127
Number of syllables (word)	2.18	0.79	1–3	1.50	0.66	1–2	6.40	< 0.001
Zipf frequency (word)	4.08	0.42	3.12–5.02	4.13	0.55	0.70–5.09	− 0.81	0.419
Phonological similarity with Italian (word)	78.41	9.86	60.48–99.64	3.84	3.11	0.88–19.72	70.67	< 0.001

Visual complexity was extracted from the MultiPic norms (Duñabeitia et al., 2018) and is expressed on a 1–5 scale. Number of syllables was extracted from N-Watch (Davis, 2005). Zipf frequency was extracted from Subtlex-UK (van Heuven et al., 2014). Phonological similarity with Italian was extracted from Spinelli and Sulpizio’s (2024) pilot study and is expressed on a 0–100 scale. A *t*-test for independent samples was conducted to compare the mean values for each characteristic for cognate and noncognate stimuli

Both the cognate set and the noncognate set were split into two subsets of 48 stimuli, roughly matched on the most relevant characteristics reported in Table 3, with one subset from each set being assigned to the low-load blocks and the other subset from each set being assigned to the high-load blocks in counterbalanced fashion (in this experiment as well, the load manipulation was administered using separate blocks, with the order of the two blocks being counterbalanced across participants but, as noted, always being consistent with the order used for the L1 Stroop experiment). All pictures were 300 pixels wide and 300 pixels high.

In addition to the picture stimuli, for the *n*-back task, the experiment involved the same 5 letters used for the L1 Stroop experiment (B, D, G, P, and T), each of which was presented an equivalent number of times across the practice and experimental trials within each load condition. The trial sequence was the same as in the L1 Stroop experiment with the exception that the picture stimulus replaced the colored stimulus and was presented for 4,000 ms or until response. All stimuli were presented in the center of the screen (with written stimuli being presented in Courier New 14 point) against a white background. Also for this task, the order of presentation of the letter and picture stimuli within a block was randomized with the constraint that the two types of stimuli be presented in an alternated fashion.

Participants received instructions structured and phrased in a fashion similar to those of the L1 Stroop experiment. For the picture-naming task, participants were instructed to name the picture in English, their L2, as quickly as possible with the name that they thought was the most appropriate. They were told to speak clearly, without hesitations, and not to worry excessively about their Italian accent. The *n*-back task, with the 0-back version in the low-load condition and the 2-back version in the high-load condition, was identical to that involved in the L1 Stroop experiment.

The practice sessions were also similar to those in the L1 Stroop experiment, i.e., with feedback for false alarms and misses on the *n*-back task. The low-load condition included nine practice trials. The high-load condition also involved nine practice trials for participants who performed the L1 Stroop experiment first, whereas for participants who performed the L2 picture naming first and had therefore no prior experience with that condition, it involved 24 practice trials. DMDX (Forster & Forster, 2003) was used to program the experiment.

##### L2-to-L1 translation task

 To assess participants’ L2 lexical fluency, we used the same L1-to-L2 translation task used by Spinelli and Sulpizio (2024). The task comprised 30 high-frequency, 30 medium-frequency, and 30 low-frequency Italian words, all of which had one (in the case of one of the words, two) acceptable English translation(s) according to Word Reference (https://www.wordreference.com/) and none of which had been involved in the previous tasks or were Italian-English cognates. Participants completed this task with no time limit in an Excel spreadsheet in which the words appeared one above the other in a fixed order of descending frequency.

#### Data analysis

Reported here are the appropriate confirmatory analyses to test the idea that both adaptive control and bilingual language production are subjected to capacity limitations. These analyses focus on the group-level results for L1 Stroop and L2 picture naming, the same type of analyses used by Kalanthroff et al. (2015). Exploratory analyses examining individual-level associations between linguistic variables and performance on L2 picture naming or L1 Stroop, and between performance across the two tasks, are reported in the Online Supplementary Materials.

For both experiments, the waveforms of vocal responses to the colored (L1 Stroop) and picture stimuli (L2 picture naming) were manually inspected with CheckVocal (Protopapas, 2007) to determine the accuracy of the response and the correct placement of timing marks. In line with our previous study (Spinelli & Sulpizio, 2024), we were lenient with the participant’s pronunciation in the L2 picture-naming experiment (e.g., with “mountain” pronounced ['mɔntain] instead of [ˈmaʊntɪn] being considered acceptable) and spelling in the L1-to-L2 translation task (e.g., with “rackoon” instead of “raccoon” being considered acceptable), but a response was considered correct only if it matched the expected response. Prior to the analyses, invalid trials due to technical failures, responses faster than 300 ms, and null responses (300 observations for L1 Stroop and 813 observations for L2 picture naming) were discarded. Prior to the latency analyses, incorrect responses (100 observations for L1 Stroop and 1619 observations for L2 picture naming) were also discarded. After discarding invalid and incorrect responses, eight participants contributed fewer than 70% of their original observations in the low-load condition (i.e., the baseline condition) in the L2 picture-naming experiment. Following a criterion determined a priori in line with previous work (Spinelli & Lupker, 2023a, 2023b; Spinelli & Sulpizio, 2024; Spinelli et al., 2020), those participants (whose original observations were 3072 for L1 Stroop and 1536 for L2 picture naming) were removed from the analyses. As a result, as noted, 48 participants remained in the final sample. Analyses with the full sample, reported in the Online Supplementary Materials, produced a similar pattern of results.

All analyses were conducted in R version 4.2.2 (R Core Team, 2022). For both experiments, accuracy performance in the *n*-back task across all participants was used to calculate sensitivity (i.e., *d'*) in the low-load (0-back task) and high-load (2-back task) conditions separately. To analyze performance in the L1 Stroop and L2 picture-naming tasks, R-default treatment contrasts were changed to sum-to-zero contrasts (i.e., contr.sum) to help interpret lower-order effects in the presence of higher-order interactions. Separate analyses were conducted for L1 Stroop and L2 picture naming. For both experiments, linear mixed-effects models were used to fit trial-level response times (RTs) and generalized linear mixed-effects models were used to fit trial-level accuracy specifying a binomial distribution with a logit link between fixed effects and the dependent variable. Also, for both experiments, tests for the fixed effects were conducted using an ANOVA with type-3 sums of squares.

Concerning random effects, the models for both experiments included random intercepts for participants and target stimuli. Analyses with the maximal random structure allowed by the data (Bates et al., 2015), reported in the Online Supplementary Materials, produced a similar pattern of results. For L1 Stroop, the fixed effects were Congruency (congruent vs. incongruent) and Load (low vs. high); for L2 picture naming, they were Cognate Status (cognate vs. noncognate) and Load (low vs. high). Analyses with Load Order (i.e., the order in which the two list types in the two load conditions were administered within an experiment) and Experiment Order (i.e., the order in which the two experiments were administered) as additional fixed effects, reported in the Online Supplementary Materials, revealed that neither order predictor modulated the key interactions between Congruency and Load (for L1 Stroop) and Cognate Status and Load (for L2 picture naming).

For the analyses reported here, in addition to the regression models described, we also obtained through backward selection the best-fitting model for RTs, as it is in that dependent variable that interactions with load typically emerge (de Fockert et al., 2001; Kalanthroff et al., 2015; Lavie et al., 2004). Further, to quantify the evidence for/against the key interaction between Load and Congruency (for L1 Stroop)/Cognate Status (for L2 picture naming), we fit two Bayesian models – an RT model with that interaction, interpreted as the alternative hypothesis *H*_*1*_, and an RT model without that interaction, interpreted as the null hypothesis *H*_*0*_. The contrast between the two models yielded *BF*_10_, with values above 1 representing evidence for the presence of the interaction and values below 1 representing evidence for the absence of the interaction (values around 1 would represent no real evidence for either hypothesis). The functions and packages used are reported in the Online Supplementary Materials.

## Results

### L1 Stroop experiment

**Fig. 1:**
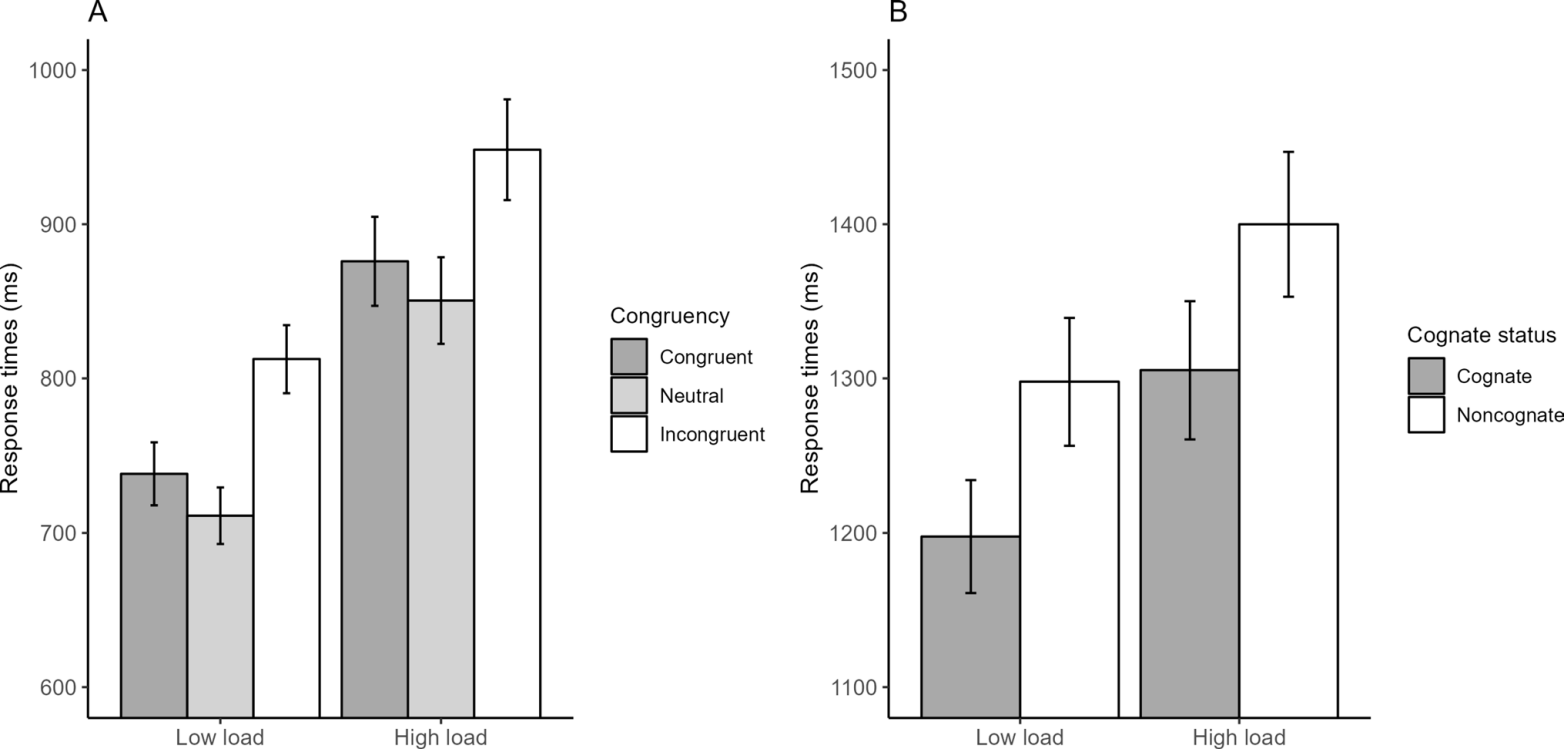
Mean participant-based response times (and corresponding 95% confidence intervals calculated using Cousineau’s (2019) method) in the L1 Stroop task (A) and the L2 picture-naming task (B)

 Concerning performance in the *n*-back task, across all participants, *d'* was 4.99 in the low-load condition (hit rate = 97.94%, false alarm rate = 0.15%), and 1.94 in the high-load condition (hit rate = 68.31%, false alarm rate = 7.04%), suggesting, as expected, higher sensitivity in the former than in the latter (although, in the high-load condition, sensitivity was still larger than zero). Concerning performance in the Stroop task, the mean participant-based RTs are presented in Fig. 1A and in Table 4 along with mean error rates. Full results from the RT and accuracy regression models are reported in the Online Supplementary Materials. Here, we focus on the results of the ANOVA. For accuracy, the only significant effect was the main effect of Congruency, χ^2^ = 118.14, *p* < 0.001, with follow-up tests revealing that incongruent stimuli were less accurate than both congruent ones (i.e., the regular Stroop effect), β = 2.112, *SE* = 0.261, *z* = 8.09, *p* < 0.001, and neutral ones (i.e., the regular interference effect), β = 2.249, *SE* = 0.278, *z* = 8.10, *p* < 0.001, whereas neutral and congruent stimuli did not differ from each other, β =  − 0.136, *SE* = 0.360, *z* =  − 0.38, *p* = 0.924 (for the main effect of Load, χ^2^ = 0.53, *p* = 0.465; for the Congruency by Load interaction, χ^2^ = 2.09, *p* = 0.351). For the RTs, there were significant main effects of Load (high slower than low), χ^2^ = 2095.61, *p* < 0.001, and Congruency, χ^2^ = 778.91, *p* < 0.001. For the latter, follow-up tests revealed that incongruent stimuli were slower than both congruent ones (i.e., the regular Stroop effect), β =  − 73.00, *SE* = 3.68, *z* =  − 19.82, *p* < 0.001, and neutral ones (i.e., the regular interference effect), β =  − 99.29, *SE* = 3.68, *z* =  − 26.96, *p* < 0.001, whereas neutral stimuli were *faster* than congruent ones (i.e., a *reverse* facilitation effect), β = 26.28, *SE* = 3.67, *z* = 7.17, *p* < 0.001. Notably, Congruency and Load did not interact, χ^2^ = 0.38, *p* = 0.826. The backward selection procedure confirmed that the additive model was the best-fitting model (see Online Supplementary Materials), and the Bayes factor, *BF*_10_ = 0.002 ± 3.61%, strongly favored the additive model over the interactive one.

### L2 picture-naming experiment

Similar to the L1 Stroop experiment, in the *n*-back task, sensitivity was higher in the low-load condition (*d'* = 5.02, hit rate = 98.05%, false alarm rate = 0.14%), than in the high-load condition (*d'* = 1.32, hit rate = 54.11%, false alarm rate = 11.16%), with sensitivity still being larger than zero in the latter condition. The mean participant-based RTs in the picture-naming task are presented in Fig. 1B and in Table 5 along with mean error rates, whereas full results from the RT and accuracy regression models are reported in the Online Supplementary Materials. Again, here, we focus on the ANOVA results. For accuracy, there were significant main effects of Load (high less accurate than low), χ2 = 4.96, *p* = 0.026, and Cognate Status (noncognate less accurate than cognate), χ2 = 6.26, *p* = 0.012, whereas the interaction was not significant, χ2 = 0.06, *p* = 0.807. Indeed, the cognate effect was numerically identical in the two load conditions (4.49%). Similarly, for the RTs, both the main effect of Load (high slower than low), χ2 = 112.70, *p* < 0.001, and that of Cognate Status (noncognate slower than cognate), χ2 = 8.23, *p* = 0.004, were significant whereas the interaction was not, χ2 = 0.09, *p* = 0.769. In this case as well, the backward selection procedure confirmed that the additive model was the best-fitting model (see Online Supplementary Materials), and the Bayes factor, BF10 = 0.040 ± 5.14%, strongly favored the additive model over the interactive one.

**Table 4:** Mean participant-based response times and percentage error rates (and corresponding 95% confidence intervals calculated using Cousineau’s (2019) method) in the L1 Stroop task

	Response times		Error rates	
Congruency	Low load	High load	Low load	High load
Congruent	738 [718, 759]	876 [847, 905]	0.30 [0.03, 0.56]	0.23 [− 0.01, 0.48]
Neutral	711 [693, 730]	850 [822, 879]	0.20 [− 0.04, 0.44]	0.26 [− 0.04, 0.57]
Incongruent	813 [791, 835]	948 [916, 981]	1.66 [0.84, 2.49]	2.68 [1.64, 3.73]
Facilitation effect (neutral – congruent)	− 27	− 26	− 0.10	0.03
Interference effect (incongruent – neutral)	102	98	1.46	2.42
Stroop effect (incongruent – congruent)	75	72	1.36	2.45

**Table 5:** Mean participant-based response times and percentage error rates (and corresponding 95% confidence intervals calculated using Cousineau’s (2019) method) in the L2 picture-naming task

	Response times		Error rates	
Cognate status	Low load	High load	Low load	High load
Cognate	1198 [1161, 1234]	1305 [1261, 1350]	10.82 [8.97, 12.68]	12.31 [10.18, 14.44]
Noncognate	1298 [1257, 1339]	1400 [1353, 1447]	15.31 [13.17, 17.44]	16.80 [13.90, 19.70]
Cognate effect	100	95	4.49	4.49

## Discussion

In the present study, neither L1 Stroop effects nor L2 picture-naming cognate effects were affected by the load created by a difficult secondary task – a result pattern that is inconsistent with most theories relevant to those effects. Theories of language-control associations, for example, assume that using a language when another is known would engage adaptive control (e.g., Bialystok, 2017). As such, processes such as bilingual language production would be subjected to similar attentional limitations as those assumed for domain-general adaptive control by a wide range of theories (e.g., Braver, 2012; Kalanthroff et al., 2018; Lavie, 2010). The fact that no evidence for such limitations emerged in either language-specific or domain-general experiments clearly calls for an explanation.

One possibility is that our paradigm was simply not sensitive enough to detect the targeted load effects. However, this possibility appears unlikely because, first, our sample size was larger than that used in most previous studies which *have* found those effects (e.g., Kalanthroff et al., 2015). Second, we used, as primary tasks, the standard tasks used to examine cognate effects and Stroop effects, and, as the secondary task, a task – the *n*-back task – which in its difficult (and proper) version (in our study, the 2-back task) undoubtedly poses heavy demands on the executive network as it involves multiple processes within it (Chatham et al., 2011), a fact demonstrated by the overall performance drop that task caused in both L1 Stroop and L2 picture naming.

Of course, it is possible that the present pattern of results may not generalize to other tasks or populations. However, since the pattern occurred in a domain-general task in addition to a language-specific one and the load manipulation applied to the two tasks was the same, it does not seem especially likely that linguistic differences would play a strong role (but note that our data revealed that bilinguals’ characteristics might be important moderating factors, as suggested, for example, by reduced load effects in L1 Stroop for highly immersed/proficient bilinguals – see Online Supplementary Materials). Future research aiming at establishing the generalizability of this pattern, therefore, would probably benefit more from focusing on variations of Stroop and concurrent tasks (see *Method* section for a few possibilities) and/or use complementary approaches in which, for example, individual differences in control abilities are also measured and put in relation with the load manipulation (e.g., Ahmed & de Fockert, 2012). An alternative idea to explore would be that the effect of a secondary task may depend more on how similar that task is to the primary task than on the amount of resources it requires (Oberauer et al., 2016).

If, however, the pattern does generalize, it would shed a new light on past (and future) studies in the relevant research programs. Concerning adaptive control, this pattern adds one more piece to the ambiguous body of evidence concerning whether selection is resource-demanding, with many studies failing to replicate the seminal findings that informational conflict is increased under load (de Fockert et al., 2001; Lavie et al., 2004) and only one study suggesting that task conflict is increased (Kalanthroff et al., 2015). However, we did not find any increase in a conceptually similar but methodologically improved replication of Kalanthroff et al.’s experiment, suggesting that a methodological confound could have contributed to their result (see *Method* section). Without that confound, reverse facilitation – a signature index of task conflict (Kalanthroff et al., 2018) – emerged in both low- and high-load conditions, suggesting that conflict was experienced to similar degrees in the two situations (see also Spinelli & Lupker, 2023b). Similar methodological differences with the seminal studies (e.g., smaller samples, the use of non-standard Stroop tasks) might explain the discrepancy with those studies. Future research should address this question through empirical (e.g., direct replication) and meta-analytical studies.

Concerning bilingualism, the literature to connect our pattern with is more scarce and less comparable (e.g., with the cognate effect being paired with unconventional load manipulations: Martin & Nozari, 2021). Interestingly, Christoffels et al. (2006) reported no significant differences in cognate effects between bilingual groups differing in working-memory capacity and, thus, the amount of resources at their disposal. This initial converging evidence, which would first and foremost require replication, would thus seem to suggest that resource availability has no bearing on the efficiency of bilingual selection.

Explaining the present findings in consideration of the past ones involves questioning the idea that selection is resource-demanding but not necessarily the idea that control, in general, *can* be adapted from proactive to reactive and vice versa, as demonstrated by several other manipulations (e.g., Spinelli et al., 2019). One possibility, therefore, is that selection may nearly always rely on reactive control under normal circumstances (i.e., with little or no load), with individuals making no special effort to prepare for conflict in advance (Braver, 2012). Therefore, even if a difficult secondary task is introduced which would make reactive control the only option, such a task would have virtually no impact on selection, i.e., on cognate or Stroop effects (while potentially having an impact on other processes such as perceptual and response-related ones, explaining overall performance drops and, potentially, other interactions reported in the literature, e.g., Soutschek et al., 2013). For the cognate effect, another possibility is that it is impervious to control manipulations, either because it reflects the result of automatic spreading activation within a specialized selection mechanism (Costa et al., 2000) or because, even if the effect involves some (controllable) interference caused by noncognates, it mostly depends on strong (uncontrollable) facilitation caused by cognates (an argument that cannot be ruled out since there is no obvious neutral condition to separate facilitation from interference in the cognate effect, and thus represents a potential limitation for the use of the effect to study adaptive control; but for evidence that facilitation, just as interference, can be adaptively controlled, see Bugg et al., 2011; Compton et al., 2012).

Importantly, for theories of language-control associations, either possibility would seem to create little potential for bilingual language production, per se, to produce more general consequences on cognition, possibly explaining the difficulty observing such consequences (Paap, 2022). The reason is that, if selection during bilingual language production mainly occurred through reactive control (or through processes granting control immunity), bilinguals would not seem to have much to gain from the additional occasions to apply that mode that bilingualism would afford. Consistent with this idea, research on potential bilingual advantages has more typically searched the advantage in proactive forms of control (e.g., Gullifer & Titone, 2021). The present results, on the other hand, suggest that an advantage, if there is one to be found, would be more likely to emerge in reactive forms of control (for some initial, albeit weak, evidence, see Spinelli et al., 2022).

As noted, such strong implications need corroboration from different instantiations of primary and secondary tasks, samples, and research groups. Note, however, that for as disruptive as they may look, the present results fit well within more general trends in the relevant literatures. Language-wise, along with Spinelli and Sulpizio’s (2024; see also, e.g., Blanco-Elorrieta & Caramazza, 2021), the present results suggest that the quest for bilingual advantages may need to take a step back (i.e., determine what exactly bilingualism might engage) in order to make two steps forward (i.e., locate reliable benefits associated with bilingualism). Control-wise, it is helpful to remember that over the years, the traditional notion of cognitive control as a set of deliberate, top-down processes (e.g., Shiffrin & Schneider, 1977) has gradually given way to more automatic, bottom-up ones (e.g., Bugg & Crump, 2012). The present results provide a further push in that direction.

## Supplementary Information

Below is the link to the electronic supplementary material.

Supplementary file1 (DOCX 0.98 MB)

## Data Availability

The datasets analyzed in the present study are available on the Open Science Framework at https://osf.io/jhcqp/.

## Code availability

The scripts used to perform the analyses in the study are available on the Open Science Framework at https://osf.io/jhcqp/.

## Declarations

### Open practices statement

The data, code, and materials for this study are publicly accessible at https://osf.io/jhcqp/. The study was not preregistered.
