# Supplementary material for: No evidence that selection is resource-demanding in conflict and bilingual language production tasks: Implications for theories of adaptive control and language-control associations
Source: Psychon Bull Rev. 2025 Mar 7;32(4):1901–14. doi: 10.3758/s13423-025-02672-y (PMC12325562; doi:10.3758/s13423-025-02672-y)
Supplement: Supplementary file 1 — Supplementary file1 (DOCX 0.98 MB) [file 13423_2025_2672_MOESM1_ESM.docx]

**Functions and packages used in mixed-effects analyses**

The lmer function in the *lmerTest* package, version 3.1-3 (Kuznetsova et al., 2017), and the glmer function in the *lme4* package, version 1.1-30 (Bates et al., 2015b), were used to fit the linear and generalized linear mixed-effects regression models, respectively, and obtain probability estimates. The Anova function in the *car* package, version 3.1-0 (Fox & Weisberg, 2019) was used to conduct ANOVAs on the regression models. The emmeans function in the *emmeans* package, version 1.7.5 (Lenth, 2022), was used to conduct follow-up analyses. The lmBF function in the *BayesFactor* package, version 0.9.12.4.4 (Morey & Rouder, 2022), was used to fit RT models with and without the key interaction between Load and Congruency (for L1 Stroop)/ Cognate Status (for L2 picture naming).

**Regression results**

The RT and accuracy regression results for the analyses reported in the main text are reported in Table S1 for the L1 Stroop task and in Table S2 for the L2 picture-naming task. Note that because we used sum contrasts, the beta for the intercept (and its associated test) represents the grand mean and the betas for the fixed effects (and their associated tests) represent deviations from the grand mean. The levels of the fixed effects which do not appear in the tables (i.e., the incongruent condition in L1 Stroop, the noncognate condition in L2 picture naming, and the high-load condition for both tasks) were those which were given a contrast weight of −1 in all contrasts.

Table S1

*Variances and standard deviations for the random effects and coefficients, standard errors, statistics, and probability values for the fixed effects used in the models of response times and accuracy in the L1 Stroop task*

|  | Response times | | | | Accuracy | | | |
| --- | --- | --- | --- | --- | --- | --- | --- | --- |
| Random effect | *Variance* | *SD* |  |  | *Variance* | *SD* |  |  |
| Participant (intercept) | 10997.48 | 104.87 |  |  | 0.475 | 0.689 |  |  |
| Color (intercept) | 24.11 | 4.91 |  |  | 0.019 | 0.138 |  |  |
| Fixed effect | β | *SE* | *t* | *p* | β | *SE* | *z* | *p* |
| Intercept | 822.55 | 15.41 | 53.39 | < .001 | 5.532 | 0.183 | 30.16 | < .001 |
| Congruency (congruent) | −15.57 | 2.12 | −7.35 | < .001 | 0.659 | 0.188 | 3.50 | < .001 |
| Congruency (neutral) | −41.86 | 2.12 | −19.75 | < .001 | 0.795 | 0.196 | 4.06 | < .001 |
| Load (low) | −68.72 | 1.50 | −45.78 | < .001 | 0.090 | 0.124 | 0.73 | .465 |
| Congruency (congruent) × Load (low) | −0.01 | 2.12 | −0.01 | .995 | −0.214 | 0.188 | −1.14 | .256 |
| Congruency (neutral) × Load (low) | −1.13 | 2.12 | −0.53 | .594 | 0.054 | 0.196 | 0.28 | .782 |

*Note.* The accuracy coefficients are in the logit scale, not in the response scale.

Table S2

*Variances and standard deviations for the random effects and coefficients, standard errors, statistics, and probability values for the fixed effects used in the models of response times and accuracy in the L2 picture-naming task*

|  | Response times | | | | Accuracy | | | | |  |
| --- | --- | --- | --- | --- | --- | --- | --- | --- | --- | --- |
| Random effect | *Variance* | *SD* |  |  | | *Variance* | *SD* |  |  | |
| Participant (intercept) | 30166.66 | 173.69 |  |  | | 0.276 | 0.525 |  |  | |
| Picture (intercept) | 46376.33 | 215.35 |  |  | | 1.685 | 1.298 |  |  | |
| Fixed effects | β | *SE* | *t* | *p* | | β | *SE* | *z* | *p* | |
| Intercept | 1326.10 | 29.95 | 44.28 | < .001 | | 2.425 | 0.131 | 18.57 | < .001 | |
| Cognate Status (cognate) | −46.99 | 16.38 | −2.87 | .005 | | 0.258 | 0.103 | 2.50 | .012 | |
| Load (low) | −54.05 | 5.09 | −10.62 | < .001 | | 0.076 | 0.034 | 2.23 | .026 | |
| Cognate Status (cognate)× Load (low) | −1.49 | 5.09 | −0.29 | .769 | | 0.008 | 0.034 | 0.244 | .807 | |

*Note.* The accuracy coefficients are in the logit scale, not in the response scale.

**Additional analyses**

Best-fitting models

For both L1 Stroop and L2 picture naming, the step function in the lmerTest package was applied to the RT models reported in the main text in order to obtain the best-fitting model using backward selection. As noted in the main text, for both tasks, the best-fitting model was that without the interaction term. Specifically, the L1 Stroop model only included the main effects of Load, χ^2^ = 2096.37, *p* < .001, and Congruency, χ^2^ = 779.12, *p* < .001. Similarly, the L2 picture naming model only included the main effects of Load, χ^2^ = 113.26, *p* < .001, and that of Cognate Status (noncognate slower than cognate), χ^2^ = 8.24, *p* = .004. In both models, the random intercepts for participants and items were retained.

Full sample

For both L1 Stroop and L2 picture naming, we conducted the same analyses reported in the main text but with no participant excluded. That is, we used the full sample of 56 participants even though some performed poorly on the L2 picture-naming task.

The mean participant-based RTs and error rates are presented in Table S3 for L1 Stroop and Table S4 for L2 picture naming. The pattern of results was exactly the same as with the sample used in the analyses reported in the main text. Specifically, for the L1 Stroop task, the only significant effect in the accuracy data was the main effect of Congruency, χ^2^ = 134.25, *p* < .001, with follow-up tests revealing the same pattern found for the analyses reported in the main text (for the main effect of Load, χ^2^ = 2.74, *p* = .098; for the Congruency by Load interaction, χ^2^ = 2.96, *p* = .228). In the RT data, there were significant main effects of Load (high slower than low), χ^2^ = 2526.13, *p* < .001, and Congruency, χ^2^ = 931.49, *p* < .001. For the latter, follow-up tests revealed, again, the same pattern found for the analyses reported in the main text. Congruency and Load did not interact in this case either, χ^2^ = 0.30, *p* = .862. The backward selection procedure confirmed that the additive model was the best-fitting model and the Bayes Factor, *BF*_10_ = 0.001 ± 21.01%, strongly favored the additive model over the interactive one.

As for the L1 Stroop task, the only significant effect for the L2 picture-naming task in the accuracy data was the main effect of Cognate Status (noncognate less accurate than cognate), χ^2^ = 8.36, *p* = .004. The main effect of Load, which was significant in the main-text analysis, was marginal here, χ^2^ = 3.59, *p* = .058. As in the main-text analysis, however, the Cognate Status by Load interaction was not significant, χ^2^ = 0.06, *p* = .799. In the RT data, as in the main-text analysis, both the main effect of Load (high slower than low), χ^2^ = 115.36, *p* < .001, and that of Cognate Status (noncognate slower than cognate), χ^2^ = 9.00, *p* = .003, were significant whereas the interaction was not, χ^2^ = 0.30, *p* = .584. The backward selection procedure confirmed that the additive model was the best-fitting model and the Bayes Factor, *BF*_10_ = .04 ±3.1%, strongly favored the additive model over the interactive one.

Table S3

*Mean participant-based response times and percentage error rates (and corresponding 95% confidence intervals calculated using Cousineau’s (2019) method) in the L1 Stroop task using the full sample*

|  | Response times | | Error rates | |
| --- | --- | --- | --- | --- |
| Congruency | Low load | High load | Low load | High load |
| Congruent | 738 [720, 756] | 879 [853, 904] | 0.34 [0.07, 0.61] | 0.29 [0.03, 0.55] |
| Neutral | 713 [697, 730] | 856 [830, 882] | 0.17 [−0.03, 0.37] | 0.34 [0.05, 0.63] |
| Incongruent | 816 [795, 836] | 956 [927, 985] | 1.62 [0.91, 2.34] | 2.82 [1.78, 3.86] |
| Facilitation effect (neutral – congruent) | −25 | −23 | −0.17 | 0.05 |
| Interference effect (incongruent – neutral) | 103 | 100 | 1.45 | 2.48 |
| Stroop effect (incongruent – congruent) | 78 | 77 | 1.28 | 2.53 |

Table S4

*Mean participant-based response times and percentage error rates (and corresponding 95% confidence intervals calculated using Cousineau’s (2019) method) in the L2 picture-naming task using the full sample*

|  | Response times | | Error rates | |
| --- | --- | --- | --- | --- |
| Cognate status | Low load | High load | Low load | High load |
| Cognate | 1210 [1174, 1245] | 1316 [1274, 1358] | 13.36 [11.49, 15.23] | 14.19 [12.33, 16.05] |
| Noncognate | 1312 [1274, 1351] | 1407 [1363, 1451] | 18.94 [16.30, 21.58] | 19.94 [17.30, 22.57] |
| Cognate effect | 102 | 91 | 5.58 | 5.75 |

Maximal random structure allowed by the data

For both L1 Stroop and L2 picture naming, we conducted the same analyses reported in the main text but specifying the maximal random structure allowed by the data (Bates et al., 2015a). That is, we first ran a model with the appropriate maximal random structure for our design (Barr et al., 2013). For L1 Stroop, the appropriate maximal random structure for our design included, for both participants and colors (i.e., the target stimuli in that task), the random intercept, the random slope of Congruency, Load, and their interaction, and the correlations between these parameters. For L2 picture naming, the appropriate maximal random structure for our design included, for participants, the random intercept, the random slope of Cognate Status, Load, and their interaction, and the correlations between these parameters; for pictures (i.e., the target stimuli in that task), the structure included the random intercept, the random slope of Load, and their correlation. In case the model with the maximal random structure did not converge, we reduced the random structure based on the results of a Principal Component Analysis (PCA) of the estimated covariance matrices for the random effects (Bates et al., 2015a). If the PCA suggested rank deficiency, we first removed the correlations among random effects. If the PCA for this zero-correlation-parameter model also suggested rank deficiency, we removed all random effects associated with zero variance components or the random effect associated with the smallest variance component, a procedure we repeated until the PCA no longer suggested rank deficiency. At this point, we reintroduced correlations among random effects and we kept them only if the PCA continued to suggest no rank deficiency. Finally, if the full-rank model still failed to converge at this stage, we applied lme4’s recommended troubleshooting procedure (see “convergence” help page in R).

For the L1 Stroop task, the maximal random structure allowed by the accuracy data included, for participants, the random intercept and the random slopes for Load and for the Congruency contrasts (but not for their interaction), and the correlations among them; for colors, it included the random intercept and the random slope for the second Congruency contrast (i.e., the contrast with 0, 1, and −1 as weights for the congruent, neutral, and incongruent conditions, respectively) and for the interaction between Load and the first Congruency contrast (i.e., the contrast with 1, 0, and −1 as weights for the congruent, neutral, and incongruent conditions, respectively). The pattern of results for the fixed effects was the same as in the analyses reported in the main text, with Congruency as the only significant effect, χ^2^ = 35.52, *p* < .001 (for Load, χ^2^ = 2.88, *p* = .090; for the Congruency by Load interaction, χ^2^ = 1.49, *p* = .474). Do note, however, that these results must be taken with caution because they come from a model which did not converge, as model convergence could not be reached even following lme4’s recommended troubleshooting procedure (we reported the results from the non-converging model nevertheless because similar results were produced by models involving other optimizers, suggesting that those convergence failures might have been false positives).

The maximal random structure allowed by the RT data included, for participants, the random intercept and all of the random slopes; for colors, it included the random intercept and all of the random slopes save for that for the first Congruency contrast (see above). The pattern of results for the fixed effects was the same as in the analyses reported in the main text, with main effects of both Load, χ^2^ = 78.38, *p* < .001, and Congruency, χ^2^ = 193.64, *p* < .001, but not their interaction, χ^2^ = 0.26, *p* = .879. Note that in this case as well, the model did not converge initially, but did converge using the BOBYQA optimizer, which is the one we reported.

For the L2 picture-naming task, the maximal random structure allowed by the accuracy data included, for participants, the random intercept, the random slopes for Load and Cognate Status (but not for their interaction), and the correlations among them; for pictures, it included the random intercept only. For the fixed effects, Cognate Status was the only significant effect, χ^2^ = 5.35, *p =* .021 (for Load, χ^2^ = 0.88, *p* = .349; for the Cognate Status by Load interaction, χ^2^ = 0.06, *p* = .810). The maximal random structure allowed by the RT data included, for participants, the random intercept and all of the random slopes; for pictures, it included the random intercept only. The pattern of results for the fixed effects was the same as in the analyses reported in the main text, with main effects of both Load, χ^2^ = 45.51, *p* < .001, and Cognate Status, χ^2^ = 7.63, *p* < .001, but not their interaction, χ^2^ = 0.04, *p* = .848.

Additional predictors

Paralleling Spinelli & Sulpizio (2024), we conducted three sets of analyses with additional predictors for exploratory purposes. In the first set, conducted for both L1 Stroop and L2 picture naming, the additional predictors were language-based measures from the LHQ3 and the L1-to-L2 translation task. In the second set, conducted for L2 picture naming only, the additional predictors were performance-based measures from the L1 Stroop task. As for Spinelli and Sulpizio (2024), these two analyses were exploratory because the present study was not set up to examine the impact of individual differences in language- or performance-based abilities. Doing so would require a sample of a larger size than the one we used, and, further, we made no attempt to produce variability in those abilities in our sample. As explained below, however, these analyses can be informative nonetheless. Finally, in the third set of analyses, conducted for both L1 Stroop naming and L2 picture naming, the additional predictors were the order in which the two tasks were administered and the order in which the two load conditions within each task were administered.

*Language-based predictors*

As in Spinelli and Sulpizio (2024), we focused on L2 measures because of their relevance to L2 picture naming and because there was little variability in L1 measures. In particular, we focused on the aggregated scores produced by the LHQ3 (i.e., proficiency, immersion, and dominance) for English (i.e., the participants’ L2) and on English lexical fluency as measured by our L1-to-L2 translation task. To avoid multicollinearity issues, we inspected the pattern of correlation between these four variables (see Table S5). Overall, the tendency was for the variables to be positively correlated with one another, as expected. The correlation between proficiency and dominance was particularly strong, however, this result is hardly surprising because proficiency is one of the variables making up the dominance score (Li et al., 2019; see also Spinelli & Sulpizio, 2024). More informative are the other correlations, with those between lexical fluency on one hand and proficiency and dominance on the other hand being weak (but significant) whereas no correlations emerged between immersion and any of the other variables. Based on these results and following Spinelli and Sulpizio (2014), we used lexical fluency and immersion as the language-based predictors in our analyses. Lexical fluency was preferred over proficiency and dominance because it is an objective measure of participants’ L2 ability, and, thus, likely less subjected to bias than the other two measures, which are based on self-ratings (Tomoschuk et al., 2019). Do note, further, that because dominance could not be calculated for two participants because of the corrections we applied (Spinelli & Sulpizio, 2024), those participants would have had to be dropped from the analysis had we picked that language-based predictor.

Table S5

*Pearson’s correlations between proficiency, immersion, dominance, and lexical fluency in English (L2)*

| Variables | 1. | 2. | 3. | 4. |
| --- | --- | --- | --- | --- |
| 1. L2 proficiency | 1 |  |  |  |
| 2. L2 immersion | .028 | 1 |  |  |
| 3. L2 dominance | .865*** | .167 | 1 |  |
| 4. L2 lexical fluency | .346* | −.066 | .301* | 1 |

*Note*. *** *p* < .001; ** *p* <.01; * *p* < .05.

What are the presumed impacts of the language-based predictors we used on performance in L1 Stroop and L2 picture naming? Concerning L1 Stroop, most hypotheses of language-control associations (e.g., Bialystok, 2017; Bialystok & Craik, 2022; Green & Abutalebi, 2013) would seem to predict that *higher* L2 immersion and/or lexical fluency should be associated with some kind of performance benefit in the Stroop task, for example, faster RTs, lower error rates, reduced congruency effects, and/or reduced load effects (e.g., Spinelli et al., 2022). On the other hand, under the controlled-dose hypothesis (Paap, 2018), this sort of domain-general advantage would be more pronounced in *earlier* stages of learning a second language, because it would be at those stages that individuals would use domain-general processes when dealing with their second language, before they develop, with greater second-language experience, language-specific processes in order to do so. As a result, the prediction would be for the performance benefits described to occur, if anything, for individuals with *lower* L2 immersion and/or lexical fluency. Be as it may, it is important to note that the verbal nature of the stimuli involved in a classic color-word Stroop task such as ours is not ideal to test either of these hypotheses, which, indeed, have most typically been tested using non-linguistic tasks (e.g., Hilchey & Klein, 2011; Hilchey et al., 2015; Paap & Greenberg, 2013).

Concerning L2 picture naming, it is generally reasonable for higher L2 immersion and/or lexical fluency to be associated with an overall processing advantage and reduced cognate effects, the pattern of results we previously observed (Spinelli & Sulpizio, 2024). Predictions concerning the impact of the load manipulation are less straightforward, with, again, most hypotheses of language-control associations (e.g., Bialystok, 2017; Bialystok & Craik, 2022; Green & Abutalebi, 2013) seeming to predict reduced load effects for individuals with *higher* L2 immersion and/or lexical fluency and the controlled-dose hypothesis (Paap, 2018) seeming to predict reduced load effects for individuals with *lower* L2 immersion and/or lexical fluency.

Note that, for both L1 Stroop and L2 picture naming, it is difficult to make predictions for the relevant three-way interaction patterns (i.e., that between Congruency, Load, and the language-based predictors for L1 Stroop, and that between Cognate Status, Load, and the language-based predictors for L2 picture naming). The reason is that those patterns would likely depend on the specific pattern of the two-way interactions between Load and the language-based predictors, a type of pattern for which, as noted, contrasting predictions can be generated.

The ideas here described were examined by including both L2 Immersion and L2 Lexical Fluency in the RT and accuracy models with their main effects and their interactions with the original predictors (i.e., for L1 Stroop, Congruency and List Type; for L2 picture naming, Cognate Status and Load). Interactions involving both Immersion and Lexical Fluency were excluded in order to reduce unnecessary model complexity. Both Immersion and Lexical Fluency were standardized before the analyses in order to help model convergence, and, if the model failed to converge anyway, we applied lme4’s recommended troubleshooting procedure (see “convergence” help page in R). The analyses were otherwise conducted as in the main text.

For L1 Stroop, the accuracy model (restarted from the apparent optimum, which converged whereas the initial model did not) only showed a significant effect of Congruency, χ^2^ = 96.96, *p* < .001, as in the main-text analysis. The only other effect approaching significance was the main effect of L2 Lexical Fluency, χ^2^ = 3.05, *p* = .081, reflecting a numerical tendency for overall higher error rates in more fluent participants (all other *p*s ≥ .117). The RT model, on the other hand, showed a few significant effects in addition to those of Load, χ^2^ = 2108.07, *p* < .001, and Congruency, χ^2^ = 783.45, *p* < .001, reported in the main-text analysis. Specifically, there was a main effect of L2 Immersion, χ^2^ = 3.88, *p* = .049, reflecting a tendency for overall faster RTs for participants with higher immersion scores (but do note the opposite numerical tendency in the accuracy data, suggesting a potential speed-accuracy trade-off). More importantly, both L2 Immersion and L2 Lexical Fluency interacted with Load, χ^2^ = 46.43, *p* < .001, and χ^2^ = 76.45, *p* < .001, respectively. Both interactions reflected a reduction of the load effect with higher scores of the language-based predictor (see Figure S1). Follow-up analyses revealed that such a pattern resulted from the fact that, whereas neither Immersion or Lexical fluency had any impact on the low-load condition (β = −19.07, *SE* = 14.94, *z* = −1.28, *p* = .202, and β = −0.80, *SE* = 14.93, *z* = −0.05, *p* = .957, respectively), they were both associated with a speed-up in the high-load condition, although such a speed-up was significant for Immersion only (β = −39.51, *SE* = 14.94, *z* = −2.64, *p* = .008; for Lexical Fluency, β = −27.04, *SE* = 14.93, *z* = −1.81, *p* = .070, respectively). No other effect was significant, all *p*s ≥ .266.

Figure S1

*The impact of L2 immersion (A) and lexical fluency (B) on RTs for the low- and high-load conditions in the L1 Stroop task*


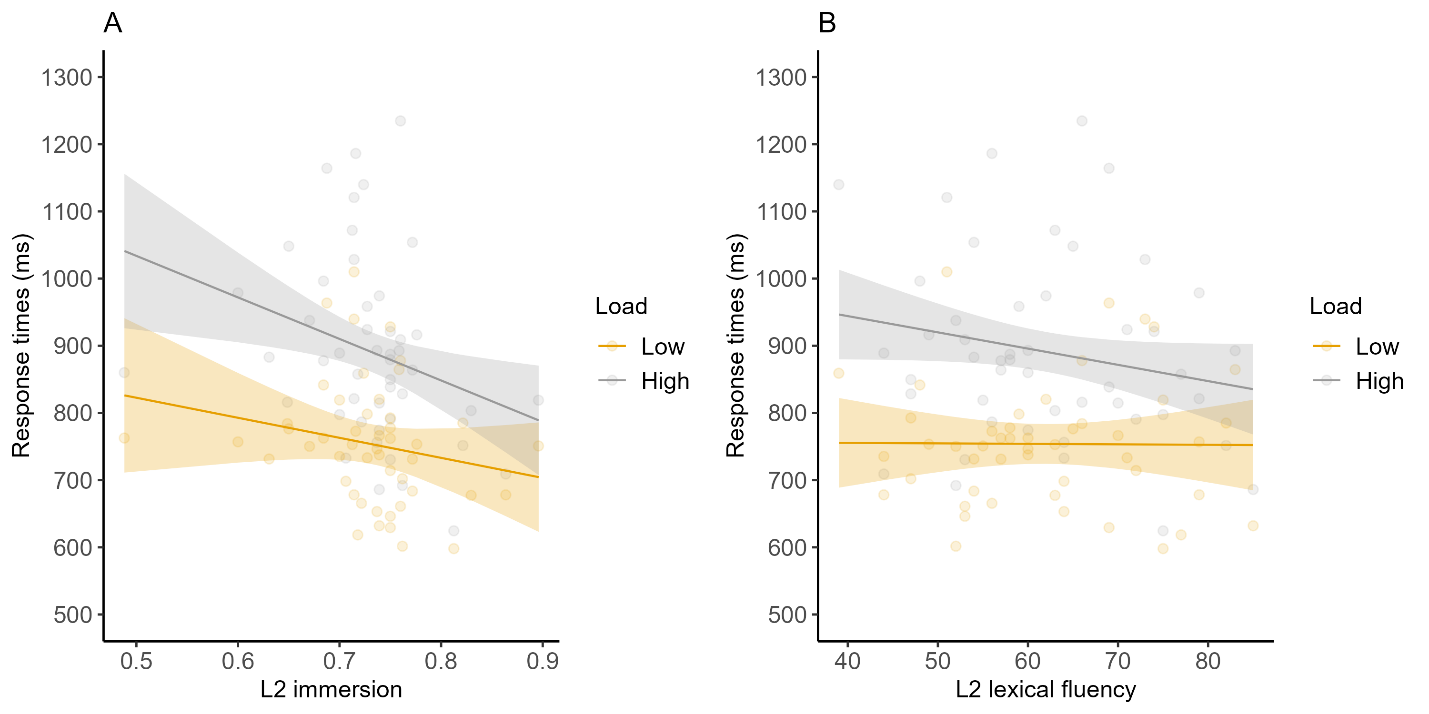
*Note.* The gold and grey circles represent mean participant RTs and the lines (with 95% confidence bands) represent the model-estimated trends for the low- and high-load conditions.

For L2 picture naming, the accuracy model (restarted from the apparent optimum, which converged whereas the initial model did not), similar to the main-text analysis, showed a significant main effect of Cognate Status, χ^2^ = 5.82, *p* = .016, and a marginal main effect of Load, χ^2^ = 3.70, *p* = .055. In addition, there was also a significant main effect of L2 Lexical Fluency, χ^2^ = 34.91, *p* < .001, reflecting lower error rates in more fluent participants. No other effect was significant, all *p*s ≥ .172. The RT model also showed the significant main effects of Cognate Status, χ^2^ = 8.30, *p* = .004, and Load, χ^2^ = 112.67, *p* < .001, reported in the main-text analysis. In addition, there was a significant main effect of L2 Lexical Fluency, χ^2^ = 38.95, *p* < .001, reflecting overall faster RTs in more fluent participants. Lexical Fluency, however, also interacted with Cognate Status, χ^2^ = 8.85, *p* = .003, with follow-up test revealing that, although both cognate and noncognate pictures were responded to faster with higher fluency scores (β = −104.16, *SE* = 19.73, *z* = −5.28, *p* < .001, and β = −134.57, *SE* = 19.86, *z* = −6.78, *p* < .001, respectively), this speed-up was more pronounced for the latter than for the former, leading to a reduction of the cognate effect with higher fluency scores (see Figure S2), the type of pattern we previously reported (Spinelli & Sulpizio, 2024).

Figure S2

*The impact of L2 lexical fluency on RTs for cognate and noncognate pictures in the L2 picture-naming task*


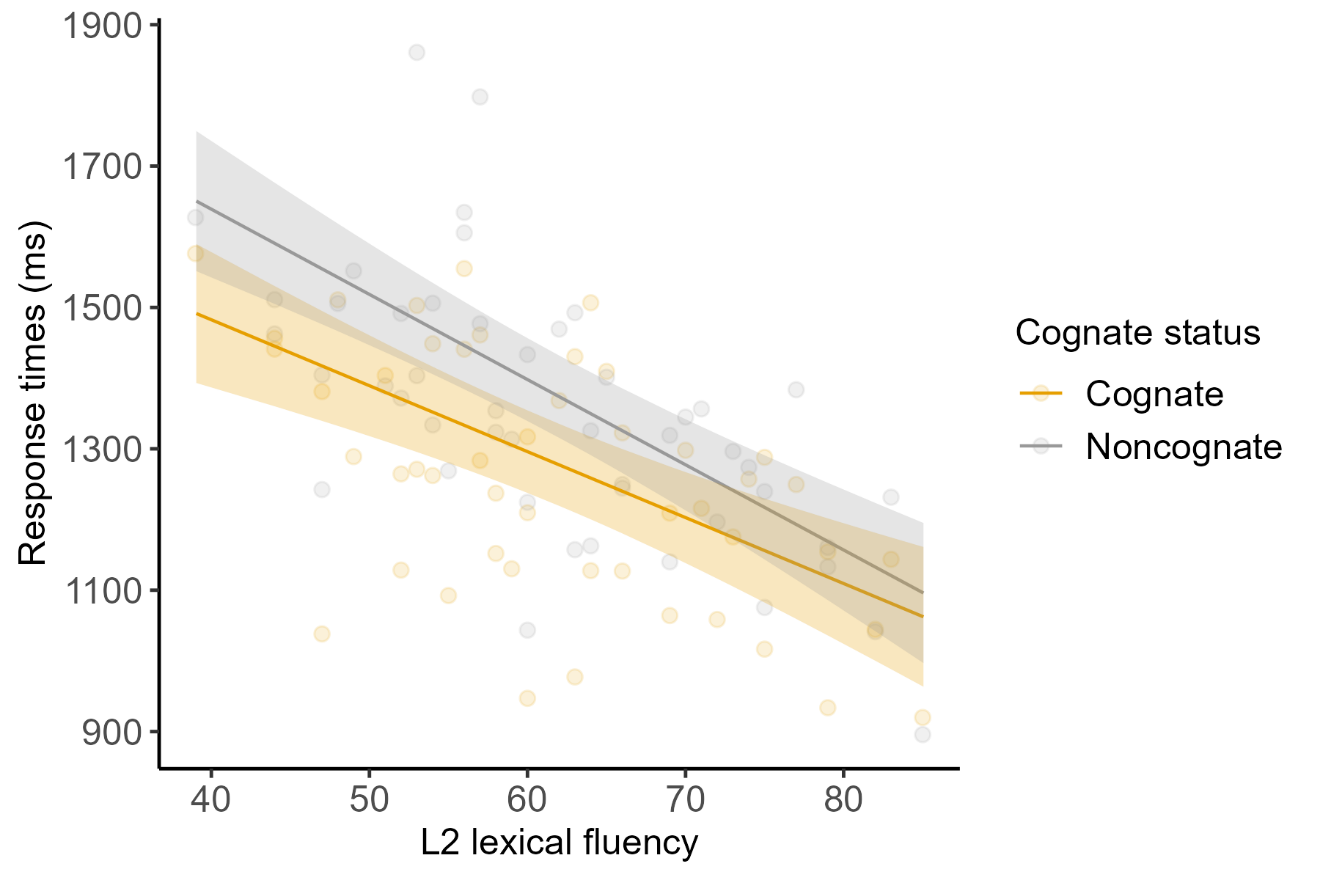


*Note.* The gold and grey circles represent mean participant RTs and the lines (with 95% confidence bands) represent the model-estimated trends for cognate and noncognate pictures.

There was also an interaction between L2 Immersion and Load, χ^2^ = 9.70, *p* = .002. This interaction, represented in Figure S3, reflected a similar pattern as the one observed for L1 Stroop: Higher immersion scores were associated with reduced load effects as a result of Immersion speeding up latencies in the high-load condition (β = −40.05, *SE* = 19.66, *z* = −2.04, *p* = .042) but not in the low-load condition (β = −8.31, *SE* = 19.62, *z* = −0.42, *p* = .672). Note, however, that there was no interaction between L2 Lexical Fluency and Load in this case, χ^2^ = 1.40, *p* = .236.

Figure S3

*The impact of L2 immersion on RTs for the low- and high-load conditions in the L2 picture-naming task*


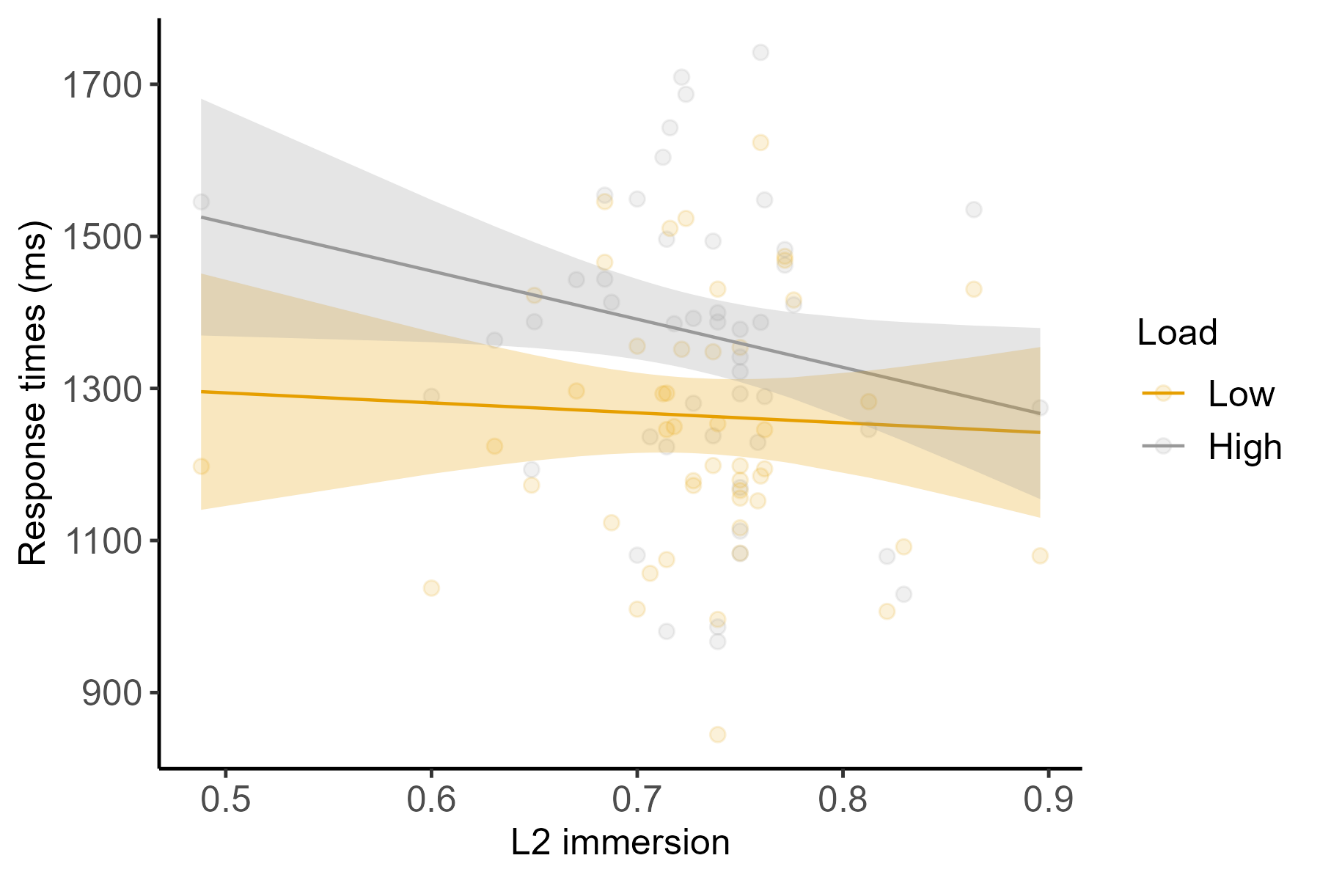
 *Note.* The gold and grey circles represent mean participant RTs and the lines (with 95% confidence bands) represent the model-estimated trends for the low- and high-load conditions.

Finally, there was a marginal three-way interaction between L2 Immersion, Load, and Cognate Status, χ^2^ = 3.33, *p* = .068, reflecting a numerical tendency for the speed-up associated with L2 Immersion to be more pronounced for cognate pictures in the low-load condition vs. *non*cognate pictures in the high-load condition. No other effect was significant, all *p*s ≥ .202.

Overall, the language-based predictors we used seemed to have an impact not only on indices directly relevant to linguistic abilities (most notably overall performance and cognate effects in L2 picture naming), as is typically observed, but also on indices not strictly language-related (most notably load effects, an impact that was observed in both L2 picture naming and, importantly, L1 Stroop). This impact was in the direction predicted by most hypotheses of language-control associations (e.g., Bialystok, 2017; Bialystok & Craik, 2022; Green & Abutalebi, 2013), theories which propose that greater experience with a second language would be conducive to domain-general performance benefits. Specifically, it is consistent with Bialystok and Craik’s (2022) idea that language-control associations might be more easily (perhaps only) observed in situations posing high demands on individuals’ attentional resources. Future research should establish whether this type of result would replicate in a more appropriate situation, i.e., an experiment involving a larger sample, a monolingual control group, and, ideally, a measure of participants’ attentional resources such as working-memory capacity as a control variable (Spinelli et al., 2022).

*Performance-based predictors*

The purpose of this analysis was to explore potential associations between performance measures on L1 Stroop and L2 picture naming. To do so, we used mixed effects modelling and reported the analysis of the L1 Stroop task as a function of performance in the L2 picture-naming task. We focused on RTs because it is in RTs that the key patterns emerged more clearly in both tasks (see also Spinelli & Sulpizio, 2024).

The additional predictors we used were the participant’s mean cognate and load effects in the L2 picture-naming task. The former is the overall difference between RTs for noncognate and cognate stimuli and can be interpreted as an index of the participant’s inability to select exclusively the target language (i.e., the larger their cognate effect overall, the worse their language selectivity; Spinelli et al., 2022; Spinelli & Sulpizio, 2024). The latter is the overall difference between RTs for the high- and low-load conditions and can be interpreted as an index of the participant’s inability to prevent a difficult secondary task from interfering with the primary task (i.e., the larger their load effect overall, the worse their ability to shield the primary task against distraction).

As with the language-based predictors, the L2 picture naming cognate and load effects were included in the RT model of L1 Stroop with their main effects and their interactions with the original L1 Stroop predictors, i.e., Congruency and Load. In this case as well, both the cognate and load effects were standardized before the analyses to help model convergence. The analysis was otherwise conducted as in the main text.

As in the main-text analysis, there were main effects of Congruency, χ^2^ = 780.46, *p* < .001, and Load, χ^2^ = 2099.04, *p* < .001. In addition, there was an interaction between Load and the L2 picture naming load effect, χ^2^ = 31.44, *p* < .001. This interaction, represented in Figure S4, reflected a positive correlation between the load effects in the two tasks, with follow-up analyses revealing that larger L2 picture naming load effects were associated with numerically slower latencies in both the low-load condition, β = 13.27, *SE* = 15.64, *z* = 0.85, *p* = .396, and especially the high-load condition, β = 30.58, *SE* = 15.64, *z* = 1.95, *p* = .051, in the L1 Stroop task.

Figure S4

*The impact of the L2 picture naming load effect on RTs for the low- and high-load conditions in the L1 Stroop task*
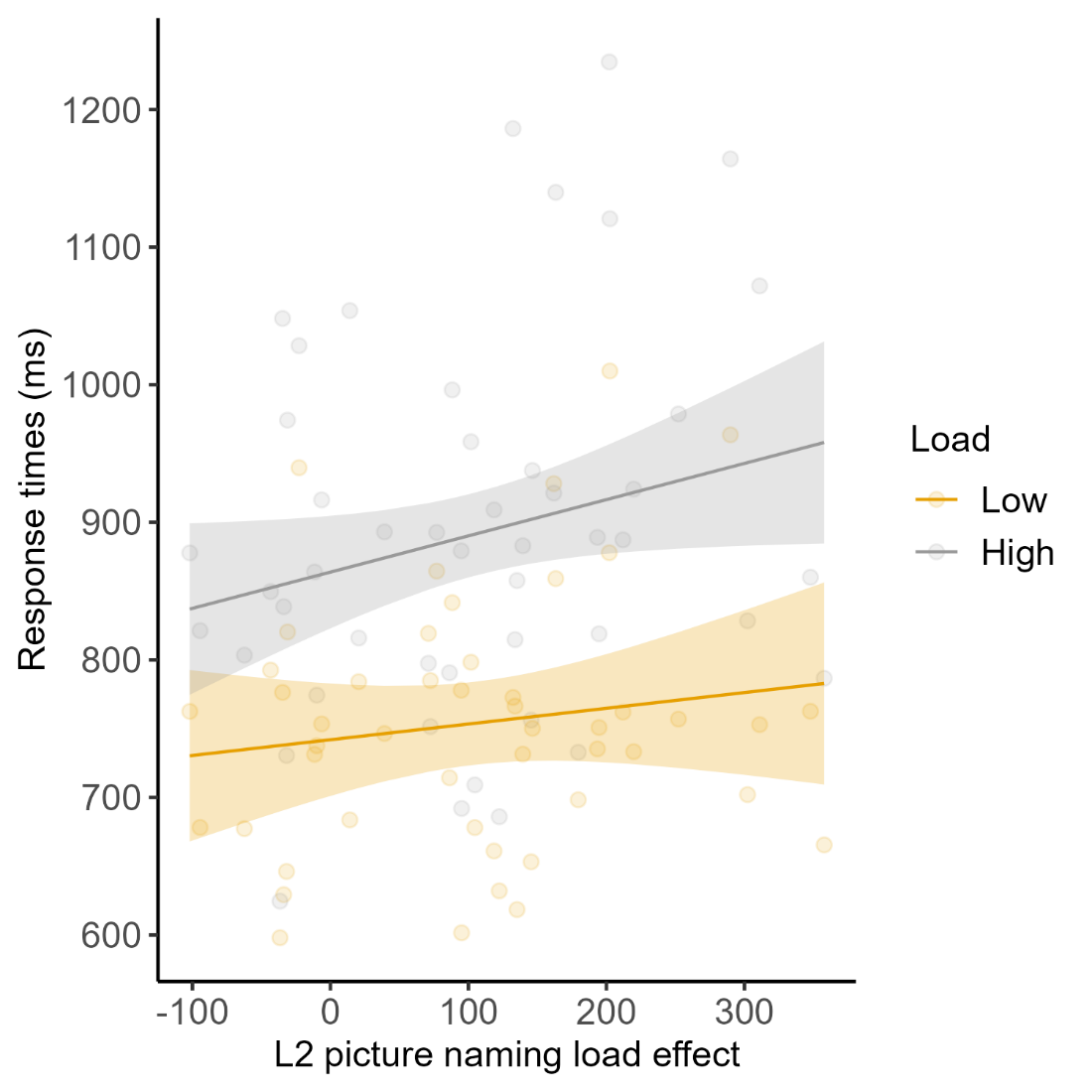
 *Note.* The gold and grey circles represent mean participant RTs and the lines (with 95% confidence bands) represent the model-estimated trends for the low- and high-load conditions.

There was also an interaction between Congruency and the L2 picture naming cognate effect, χ^2^ = 7.58, *p =* .026. Follow-up analyses revealed that the source of this interaction was the fact that larger L2 picture naming cognate effects were associated with larger L1 Stroop *interference* effects, i.e., a larger difference between incongruent and neutral stimuli, β = −10.32, *SE* = 3.78, *z* = −2.73, *p* = .017. In other words, L2 picture naming cognate effects and L1 Stroop interference effects were positively correlated. Neither the facilitation effect (i.e., neutral – congruent) nor the Stroop effect (i.e., incongruent – congruent) were affected by L2 picture naming, β = 3.93, *SE* = 3.77, *z* = 1.04, *p* = .551, and β = −6.39, *SE* = 3.79, *z* = −1.69, *p* = .211, respectively.

This two-way interaction, however, was qualified by a three-way interaction between Congruency, the L2 picture naming cognate effect, and *Load*, χ^2^ = 7.94, *p =* .019. Follow-up analyses revealed that the pattern described above for the two-way interaction between Congruency and the L2 picture naming cognate effect, i.e., larger L2 picture naming cognate effects being associated with larger L1 Stroop interference effects, only held in the high-load condition, β = −19.98, *SE* = 5.36, *z* = −3.73, *p* = .001. In addition, in that condition, larger L2 picture naming cognate effects were also associated with larger facilitation effects, β = 12.59, *SE* = 5.34, *z* = 2.36, *p* = .048. In contrast, there was no association between L2 picture naming cognate effects and Stroop effects, β = −7.40, *SE* = 5.38, *z* = −1.37, *p* = .354. Indeed, as represented in Figure S5B, the main reason interference and facilitation effects increased with larger L2 picture naming cognate effects in the high-load condition was the neutral stimuli speeding up with larger L2 picture naming cognate effects (see the grey line going down) whereas the congruent (gold line) and incongruent stimuli (light blue line) remained essentially unaffected (but do note that the slope of neither line was significantly different from zero, all *p*s ≥ 330). In contrast, in the low-load condition, represented in Figure S5A, there was no association between L2 picture naming cognate effects and either interference, facilitation, or Stroop effects, all *p*s ≥ 571.

No other effect was significant, all *p*s ≥ 159.

Figure S5

*The impact of the L2 picture naming cognate effect on RTs for congruent, neutral, and incongruent stimuli in the low- (A) and high-load (B) conditions in the L1 Stroop task
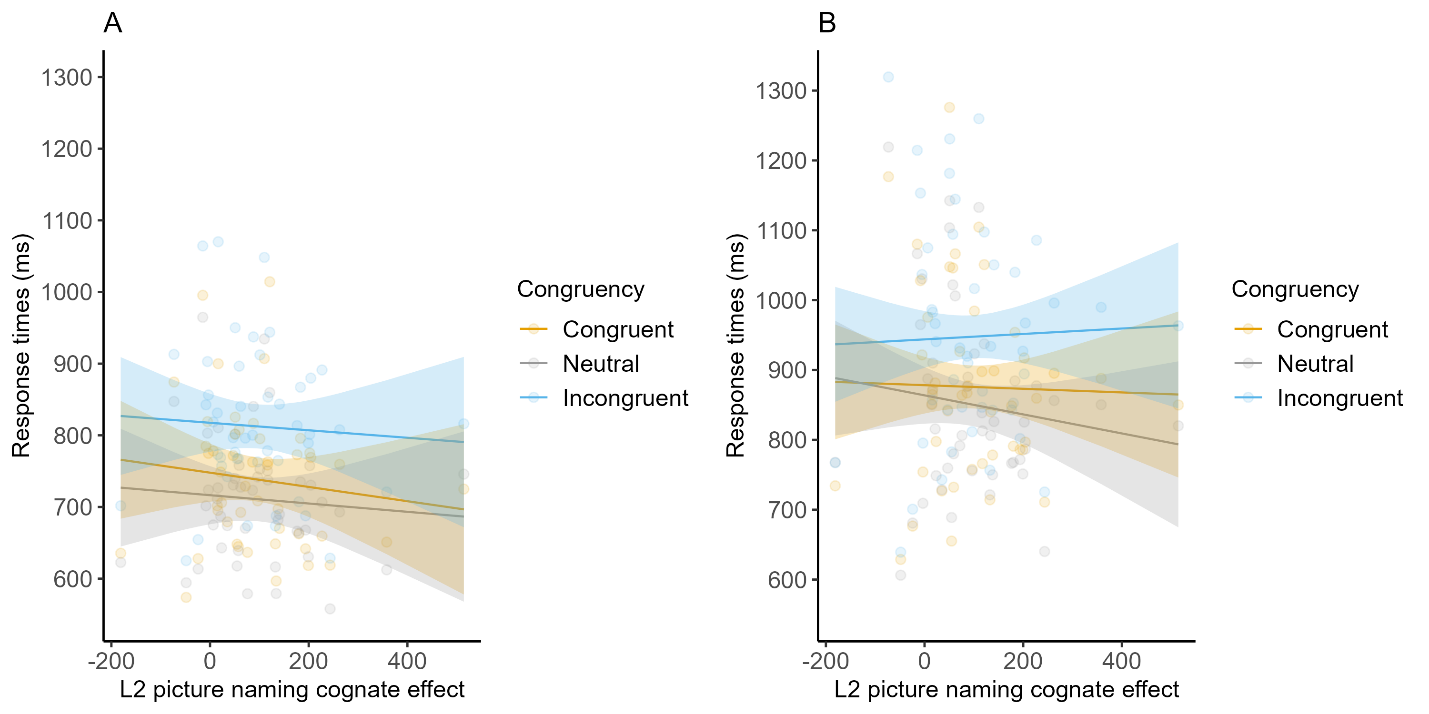
Note.* The gold, grey, and light blue circles represent mean participant RTs and the lines (with 95% confidence bands) represent the model-estimated trends for congruent, neutral, and incongruent stimuli, respectively.

Overall, this analysis revealed a few positive associations between performance in L1 Stroop and L2 picture naming. First, load effects in the two tasks were positively correlated: Participants who suffered less/more from the difficult secondary task in performing the L1 Stroop task suffered less/more from the difficult secondary task in performing the L2 picture-naming task as well. Because the secondary task was the same in the two tasks (i.e., a *n*-back task), this result is reassuring but not particularly surprising.

More interesting is the fact that L2 picture naming cognate effects were positively associated with L1 Stroop facilitation and interference effects, although only in the high-load condition of the L1 Stroop task. This type of result would seem to be consistent with theories of language associations (e.g., Bialystok, 2017; Bialystok & Craik, 2022; Green & Abutalebi, 2013) which assume positive associations between linguistic abilities such as language selectivity (which cognate effects might be taken to reflect) and control abilities such as distractor selectivity (which interference and facilitation effects in L1 Stroop might be taken to reflect). However, a couple of facts invite caution. First, as noted, the positive association in question was observed only in one of the load conditions and was mainly driven by a speed-up of the neutral stimuli with larger cognate effects. Because those stimuli should involve neither facilitation nor interference, it is unclear why they should be associated with participants’ ability to selectively attend to the target language (for similar reasoning, see also Paap et al., 2015). Second, we previously observed a *negative* association between L2 cognate effects and L1 Stroop effects in Spinelli and Sulpizio (2024). That study did not allow Stroop effects to be separated into facilitation and interference components (i.e., only congruent and incongruent stimuli were present), nor did it involve a load manipulation. However, that study did involve the same picture stimuli in the L2 picture-naming task and, because it involved no secondary task, it can be assumed that the situation it created was analogous to the low-load condition in the present study. Therefore, it is unclear why the negative association between L2 cognate effects and L1 Stroop effects which emerged in that study did not emerge in the low-load condition in the present study. At this point, it would seem safe to conclude that the association between L2 cognate effects and L1 Stroop effects (and their interference and facilitation components) is not particularly stable, and further research is needed in order to establish the existence and the direction of that association, as well as its potential moderating factors.

*Order predictors*

The present analyses were conducted to determine, for both L1 Stroop and L2 picture naming, whether the key interaction between Load and Congruency (for L1 Stroop)/Cognate Status (for L2 picture naming) might have been modulated by the order in which participants received the two experiments and the two load conditions within each experiment (but do note that both task and load orders were counterbalanced across participants, even though load order was always compatible across the two tasks, e.g., participants presented with the low-load condition first in L1 Stroop were always presented with the low-load condition first in L2 picture naming). To that aim, the main-text analyses were repeated with Experiment Order (L1 Stroop first vs. L2 picture naming first) and Load Order (low-load first vs. high-load first) as additional predictors with all potential interactions.

For L1 Stroop, the mean participant-based RTs and error rates are presented in Table S6. Note that, for the error rates, the 95% confidence intervals could not be calculated for most of the groups (i.e., all except for participants who performed the L2 picture naming first and high-load condition first) because there was no variability in one or more of the cells of the design in those groups (i.e., no errors were committed in those cells). The results of the ANOVA for the fixed effects are presented in Table S7 (the results reported for the accuracy model were obtained using the Nelder Mead optimizer, the only one that converged). Here, for simplicity, we refrain from a thorough discussion of effects which, albeit significant, have little theoretical value (e.g., practice/fatigue effects). Instead, we focus on the interaction between Load and Congruency and its higher-order interactions (i.e., the three-way interaction between Load, Congruency, and Experiment Order, the three-way interaction between Load, Congruency, and Load Order, and the four-way interaction between Load, Congruency, Experiment Order, and Load Order). As can be seen from Table S7, none of those interactions was significant in either the RT or accuracy data.

Table S6

*Mean participant-based response times and percentage error rates (and corresponding 95% confidence intervals calculated using Cousineau’s (2019) method) in the L1 Stroop task, for participants who received that task first vs. second and for participants who received the low-load condition first vs. the high-load condition first*

|  | Response times | | Error rates | |
| --- | --- | --- | --- | --- |
| Congruency | Low load | High load | Low load | High load |
| L1 Stroop first |  |  |  |  |
| Low-load first |  |  |  |  |
| Congruent | 756 [708, 804] | 933 [850, 1017] | 0.40 | 0.13 |
| Neutral | 719 [670, 767] | 912 [836, 988] | 0.13 | 0.00 |
| Incongruent | 822 [775, 868] | 1013 [937, 1089] | 0.92 | 2.44 |
| Facilitation effect (neutral – congruent) | –37 | –21 | –0.27 | –0.13 |
| Interference effect (incongruent – neutral) | 103 | 101 | 0.79 | 2.44 |
| Stroop effect (incongruent – congruent) | 66 | 80 | 0.52 | 2.31 |
| High-load first |  |  |  |  |
| Congruent | 697 [669, 726] | 817 [779, 856] | 0.53 | 0.00 |
| Neutral | 683 [657, 708] | 778 [742, 815] | 0.41 | 0.66 |
| Incongruent | 777 [741, 812] | 883 [833, 933] | 1.61 | 2.63 |
| Facilitation effect (neutral – congruent) | –14 | –39 | –0.12 | 0.66 |
| Interference effect (incongruent – neutral) | 94 | 105 | 1.20 | 1.97 |
| Stroop effect (incongruent – congruent) | 80 | 66 | 1.08 | 2.63 |
| L2 picture naming first |  |  |  |  |
| Low-load first |  |  |  |  |
| Congruent | 756 [724, 789] | 834 [792, 875] | 0.00 | 0.27 |
| Neutral | 733 [703, 763] | 823 [778, 867] | 0.00 | 0.14 |
| Incongruent | 823 [775, 870] | 884 [830, 937] | 1.06 | 2.89 |
| Facilitation effect (neutral – congruent) | –23 | –11 | 0.00 | –0.13 |
| Interference effect (incongruent – neutral) | 90 | 61 | 1.06 | 2.75 |
| Stroop effect (incongruent – congruent) | 67 | 50 | 1.06 | 2.62 |
| High-load first |  |  |  |  |
| Congruent | 743 [686, 800] | 919 [864, 975] | 0.26 [–0.26, 0.78] | 0.54 [–0.36, 1.43] |
| Neutral | 710 [664, 756] | 889 [835, 944] | 0.26 [–0.26, 0.78] | 0.26 [–0.51, 1.03] |
| Incongruent | 829 [781, 878] | 1014 [946, 1082] | 3.06 [0.82, 5.29] | 2.77 [–0.32, 5.87] |
| Facilitation effect (neutral – congruent) | –33 | –30 | 0 | –0.28 |
| Interference effect (incongruent – neutral) | 119 | 125 | 2.8 | 2.51 |
| Stroop effect (incongruent – congruent) | 86 | 95 | 2.8 | 2.23 |

Table S7

*ANOVA results for the fixed effects for response times and accuracy in the L1 Stroop task*

|  | *Response times* | | *Accuracy* | |
| --- | --- | --- | --- | --- |
| Fixed effect | χ^2^ | *p* | χ^2^ | *p* |
| Intercept | 2994.85 | < .001 | 1.18 | .277 |
| Congruency | 792.16 | < .001 | 0.30 | .863 |
| Load | 2128.80 | < .001 | < 0.01 | .992 |
| Load Order | 0.56 | .454 | 0.04 | .842 |
| Experiment Order | 0.23 | .634 | < 0.01 | 1 |
| Congruency × Load | 0.35 | .838 | < 0.01 | .999 |
| Congruency × Load Order | 10.10 | .006 | 0.12 | .940 |
| Load × Load Order | 4.62 | .032 | 0.04 | .840 |
| Congruency × Experiment Order | 0.30 | .859 | < 0.01 | 1 |
| Load × Experiment Order | 10.06 | .002 | 0.12 | .726 |
| Load Order × Experiment Order | 4.66 | .031 | 0.04 | .847 |
| Congruency × Load × Load Order | 3.65 | .161 | 0.07 | .968 |
| Congruency × Load × Experiment Order | 1.21 | .546 | 0.13 | .937 |
| Congruency × Load Order × Experiment Order | 11.43 | .003 | 0.08 | .963 |
| Load × Load Order × Experiment Order | 236.88 | < .001 | 0.03 | .870 |
| Congruency × Load × Load Order × Experiment Order | 3.18 | .204 | 0.11 | .948 |

For L2 picture naming, the mean participant-based RTs and error rates are presented in Table S8 and the results of the ANOVA for the fixed effects are presented in Table S9 (the accuracy results reported are from the model restarted from the apparent optimum, which converged whereas the initial model did not). Similar to what was done for the L1 Stroop task, for simplicity, we focus on the interaction between Load and Cognate Status and its higher-order interactions (i.e., the three-way interaction between Load, Cognate Status, and Experiment Order, the three-way interaction between Load, Cognate Status, and Load Order, and the four-way interaction between Load, Cognate Status, Experiment Order, and Load Order). In this case as well, as can be seen from Table S9, none of those interactions was significant in either the RT or accuracy data.

In sum, there was no suggestion in our data that the key interactions between Congruency and Load (for L1 Stroop) and Cognate Status and Load (for L2 picture naming) were modulated by the order in which participants received either the two experiments or the load conditions within each experiment.

Table S8

*Mean participant-based response times and percentage error rates (and corresponding 95% confidence intervals calculated using Cousineau’s (2019) method) in the L2 picture-naming task, for participants who received that task first vs. second and for participants who received the low-load condition first vs. the high-load condition first*

|  | Response times | | Error rates | |
| --- | --- | --- | --- | --- |
| Congruency | Low load | High load | Low load | High load |
| L1 Stroop first |  |  |  |  |
| Low-load first |  |  |  |  |
| Cognate | 1315 [1248, 1383] | 1410 [1321, 1498] | 12.14 [ 8.38, 15.91] | 13.74 [ 9.04, 18.45] |
| Noncognate | 1376 [1286, 1466] | 1520 [1434, 1605] | 18.82 [13.51, 24.13] | 20.85 [13.92, 27.78] |
| Cognate effect (noncognate – cognate) | 61 | 110 | 6.68 | 7.11 |
| High-load first |  |  |  |  |
| Cognate | 1136 [1065, 1208] | 1161 [1096, 1226] | 9.15 [ 6.39, 11.90] | 15.90 [11.21, 20.59] |
| Noncognate | 1325 [1236, 1414] | 1353 [1246, 1460] | 17.32 [13.86, 20.78] | 20.23 [15.01, 25.45] |
| Cognate effect (noncognate – cognate) | 189 | 192 | 8.17 | 4.33 |
| L2 picture naming first |  |  |  |  |
| Low-load first |  |  |  |  |
| Cognate | 1129 [1066, 1191] | 1332 [1251, 1414] | 10.71 [5.26, 16.17] | 8.80 [5.78, 11.82] |
| Noncognate | 1211 [1132, 1291] | 1359 [1265, 1454] | 13.50 [8.83, 18.17] | 12.19 [6.14, 18.23] |
| Cognate effect (noncognate – cognate) | 82 | 27 | 2.79 | 3.39 |
| High-load first |  |  |  |  |
| Cognate | 1210 [1131, 1290] | 1318 [1210, 1427] | 11.30 [6.96, 15.63] | 10.81 [6.14, 15.48] |
| Noncognate | 1279 [1206, 1352] | 1368 [1282, 1454] | 11.58 [8.09, 15.08] | 13.92 [8.70, 19.15] |
| Cognate effect (noncognate – cognate) | 69 | 50 | 0.28 | 3.11 |

Table S9

*ANOVA results for the fixed effects for response times and accuracy in the L2 picture-naming task*

|  | *Response times* | | *Accuracy* | |
| --- | --- | --- | --- | --- |
| Fixed effect | χ^2^ | *p* | χ^2^ | *p* |
| Intercept | 2094.70 | < .001 | 370.24 | < .001 |
| Cognate Status | 8.59 | .003 | 6.05 | .014 |
| Load | 110.86 | < .001 | 4.21 | .040 |
| Load Order | 1.80 | .179 | < 0.01 | .952 |
| Experiment Order | 1.40 | .237 | 7.54 | .006 |
| Cognate Status × Load | 0.05 | .816 | 0.02 | .893 |
| Cognate Status × Load Order | 7.20 | .007 | 0.60 | .438 |
| Load × Load Order | 14.83 | < .001 | 4.53 | .033 |
| Cognate Status × Experiment Order | 16.65 | < .001 | 5.37 | .020 |
| Load × Experiment Order | 9.47 | .002 | 7.17 | .007 |
| Load Order × Experiment Order | 4.25 | .039 | 0.27 | .605 |
| Cognate Status × Load × Load Order | < 0.01 | .974 | 0.45 | .501 |
| Cognate Status × Load × Experiment Order | 1.10 | .294 | 2.45 | .117 |
| Cognate Status × Load Order × Experiment Order | 5.56 | .018 | 0.37 | .546 |
| Load × Load Order × Experiment Order | 0.02 | .900 | 0.02 | .890 |
| Cognate Status × Load × Load Order × Experiment Order | 0.85 | .357 | 1.82 | .177 |

**References (not already included in the main text’s reference list)**

Barr, D. J., Levy, R., Scheepers, C., & Tily, H. J. (2013). Random effects structure for confirmatory hypothesis testing: Keep it maximal. *Journal of Memory and Language*, *68*, 255–278. https://doi.org/10.1016/j.jml.2012.11.001

Bates, D., Kliegl, R., Vasishth, S., & Baayen, H. (2015a). Parsimonious mixed models. *ArXiv*, 1506.04967. https://doi.org/10.48550/arXiv.1506.04967

Bates, D., Mächler, M., Bolker, B., & Walker, S. (2015b). Fitting linear mixed-effects models using lme4. *Journal of Statistical Software*, *67*, 1–48. https://doi.org/10.18637/jss.v067.i01

Bialystok, E., & Craik, F. I. M. (2022). How does bilingualism modify cognitive function? Attention to the mechanism. *Psychonomic Bulletin & Review*, *29*(4), 1246–1269. https://doi.org/10.3758/s13423-022-02057-5

Fox, J. & Weisberg, S. (2019). *An R companion to applied regression*. Thousand Oaks, CA: Sage. http://socserv.socsci.mcmaster.ca/jfox/Books/Companion

Hilchey, M. D., & Klein, R. M. (2011). Are there bilingual advantages on nonlinguistic interference tasks? Implications for the plasticity of executive control processes. *Psychonomic Bulletin & Review*, *18*(4), 625–658. https://doi.org/10.3758/s13423-011-0116-7

Hilchey, M. D., Saint-Aubin, J. & Klein, R. M. (2015) Does bilingual exercise enhance cognitive fitness in traditional non-linguistic executive processing tasks? In J. W. Schwieter (Ed.), *The Cambridge handbook of bilingual processing* (pp. 586-613). Cambridge, UK: Cambridge University Press. https://doi.org/10.1017/cbo9781107447257.026

Kuznetsova, A., Brockhoff, P. B., & Christensen, R. H. B. (2017). lmerTest Package: Tests in Linear Mixed Effects Models. *Journal of Statistical Software*, *82*(13), 1–26. https://doi.org/10.18637/jss.v082.i13

Lenth, R. (2022). Emmeans: Estimated Marginal Means, aka Least-Squares Means. R package version 1.7.5. https://CRAN.R-project.org/package=emmeans

Morey, R., & Rouder, J. (2022). BayesFactor: Computation of Bayes Factors for common designs. R package version 0.9.12-4.4. https://CRAN.R-project.org/package=BayesFactor

Paap, K. R. (2018). Bilingualism in cognitive science: The characteristics and consequences of bilingual language control. In A. De Houwer & L. Ortega (Eds.), *The Cambridge handbook of bilingualism* (pp. 435– 465). Cambridge, UK: Cambridge University Press. https://doi.org/10.1017/9781316831922.023

Paap, K. R., Johnson, H. A., & Sawi, O. (2015). Bilingual advantages in executive functioning either do not exist or are restricted to very specific and undetermined circumstances. *Cortex*, *69*, 265–278. https://doi.org/10.1016/j.cortex.2015.04.014

Tomoschuk, B., Ferreira, V. S., & Gollan, T. H. (2019). When a seven is not a seven: Self-ratings of bilingual language proficiency differ between and within language populations. *Bilingualism: Language and Cognition*, *22*(3), 516–536. https://doi.org/10.1017/s1366728918000421
